# A hardware-implemented adaptive pruning neural network

**DOI:** 10.1093/nsr/nwag547

**Published:** 2026-08-27

**Authors:** Yusen Tian, Penghao Chen, Ziyu Ming, Zengxing Zhang

**Affiliations:** School of Microelectronics, Fudan University, Shanghai 200433, China; School of Microelectronics, Fudan University, Shanghai 200433, China; School of Microelectronics, Fudan University, Shanghai 200433, China; School of Microelectronics, Fudan University, Shanghai 200433, China

**Keywords:** brain-inspired hardware, adaptive pruning neural network, ferroelectric memory, ambipolar transport, CMOS-compatible process

## Abstract

With the rapid growth of artificial intelligence (AI) applications, there is an urgent demand for edge computing hardware with low latency and high energy efficiency. The human brain, a highly parallel and sparsely connected architecture, can efficiently process complex tasks at exceptionally low power consumption, a capability that critically relies on synaptic pruning during neural development. However, current neuromorphic hardware, despite its capability to emulate the human brain for AI tasks, lacks an inherent mechanism to assess the importance of synaptic connections and thus cannot achieve brain-like adaptive synaptic pruning. Here, we demonstrate an adaptive pruning convolutional neural network based on Si-based ferroelectric ambipolar field-effect transistor (Fe-AmFET) crossbar arrays. The reconfigurable ambipolar transport in the Si Fe-AmFET memories enables a single device to implement both positive and negative synaptic weights while intrinsically evaluating connection importance, thereby enabling the crossbar arrays to perform multiply-accumulate (MAC) operations, nonvolatile weight storage and *in situ* kernel pruning within the network. Our studies indicate that the network achieves an ultrahigh kernel pruning ratio of 89.58% with negligible accuracy degradation on the Fashion-MNIST dataset, while reducing inference parameters, MAC operations and core power consumption (including the array and readout circuits) by 92.2%, 97.5% and 92.4%, respectively.

## Teaser

Brain-inspired adaptive pruning convolutional neural network hardware is realized using Si ferroelectric ambipolar field-effect transistor crossbar arrays for energy-efficient neuromorphic computing.

## INTRODUCTION

Owing to its massively parallel architecture and highly sparse interconnectivity, the human brain can accomplish a wide range of complex tasks with exceptional energy efficiency [1,2]. Within this neural system, information transmission relies on excitatory or inhibitory neurotransmitters released by synapses [3,4]. To maximize operational efficiency, the brain undergoes synaptic pruning, a structural optimization process that primarily occurs during neural development [5–7]. By continuously assessing the functional importance of each synapse, redundant synapses with low functional significance are physically eliminated. This cuts down the number of active participants in the neural system while maintaining a sparse yet highly efficient configuration (Fig. 1a). Inspired by this sophisticated neural mechanism of the human brain, various neuromorphic hardware architectures, such as in-memory computing (IMC) systems based on memristor arrays, have been successfully developed to handle artificial intelligence (AI) workloads [8–21]. However, such hardware lacks a native physical mechanism to distinguish critical synapse devices from redundant ones and thus cannot assess the importance of the synaptic connections. As a result, synaptic pruning has to be performed via offline external software processing to adapt to diverse task requirements. This leads to intolerable energy inefficiency, especially for edge computing applications.

**Figure 1.**
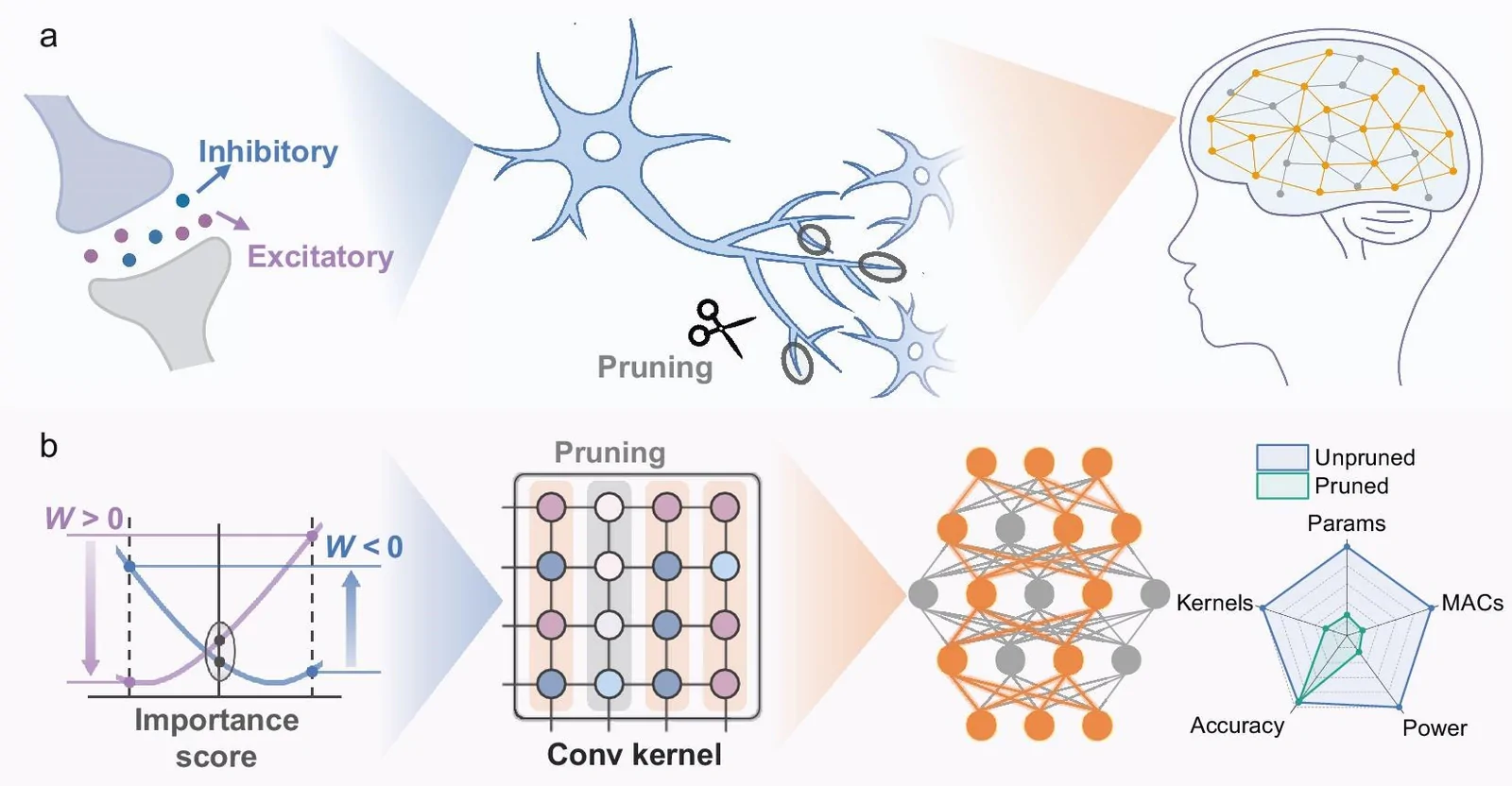
Brain-inspired adaptive pruning neural network. (a) Illustration of synaptic pruning in the human brain. During neural development, redundant biological synapses are selectively pruned, resulting in sparse synaptic connections that significantly improve the brain’s energy efficiency while preserving functional integrity. (b) Schematic of the Si Fe-AmFET-based adaptive pruning neural network. Leveraging the ambipolar memory characteristics of the Fe-AmFETs, artificial synaptic devices behave with reconfigurable positive and negative weights as well as an inherent capability to evaluate connection importance, thereby enabling adaptive pruning directly within crossbar arrays.

Herein, we report an adaptive pruning convolutional neural network (APCNN) built on Si ferroelectric ambipolar field-effect transistor (Fe-AmFET) memories (Fig. 1b), whose fabrication process is compatible with complementary metal-oxide-semiconductor (CMOS) technology. Benefitting from the reconfigurable ambipolar transport of electrons and holes in the Si Fe-AmFETs, a single Fe-AmFET is capable of achieving both positive and negative synaptic weights; meanwhile, the output current at zero gate voltage can serve as a metric for evaluating the importance of weights. The latter feature enables hardware-intrinsic assessment of connection importance without reliance on external software processing. Leveraging these unique device characteristics, we design Si Fe-AmFET crossbar arrays for the APCNN hardware, integrating multiple functions of multiply-accumulate (MAC) operations, nonvolatile weight storage, and the identification and pruning of redundant kernels. This hardware architecture can perform adaptive structural optimization of the CNN with only negligible degradation in inference accuracy. Our work should offer a feasible pathway toward low-power, high-performance embedded AI hardware, effectively addressing the energy-efficiency challenges in edge computing applications.

## RESULTS

### Weight programming and importance evaluation in the Fe-AmFETs

Figure 2a schematically illustrates the device structure and fabrication flow of the Si Fe-AmFETs, as detailed in Fig. S6. Fabrication of the devices is based on silicon-on-insulator (SOI) wafers and is compatible with CMOS technology, enabling potential for low-cost, wafer-scale manufacture [22]. An ambipolar ohmic contact, comprising complementary *p*^++^ and *n*^++^ subregions, serves as the source and drain to facilitate efficient transport of both electrons and holes. Different from conventional Schottky contacts widely employed in ambipolar transistors, the current across the ambipolar ohmic contact is dominated by quantum tunneling [22,23]. As a result, the transport is negligibly influenced by the work functions of the contacting metals. In our devices, a ferroelectric Hf_0.5_Zr_0.5_O_2_ (HZO) film is adopted as the dielectric layer, with the ferroelectric properties confirmed by positive-up-negative-down characterizations (Fig. S7) [24–29]. Figure 2b shows an optical microscopy image of a device array, and the inset displays a scanning electron microscopy (SEM) image of a typical device, which shows that the device features a gate length of 5 μm and a channel width of 20 μm. Notably, our studies indicate that the Si Fe-AmFETs exhibit considerable downscaling potential, which is significant for high-density integration in future hardware systems (Fig. S8).

**Figure 2.**
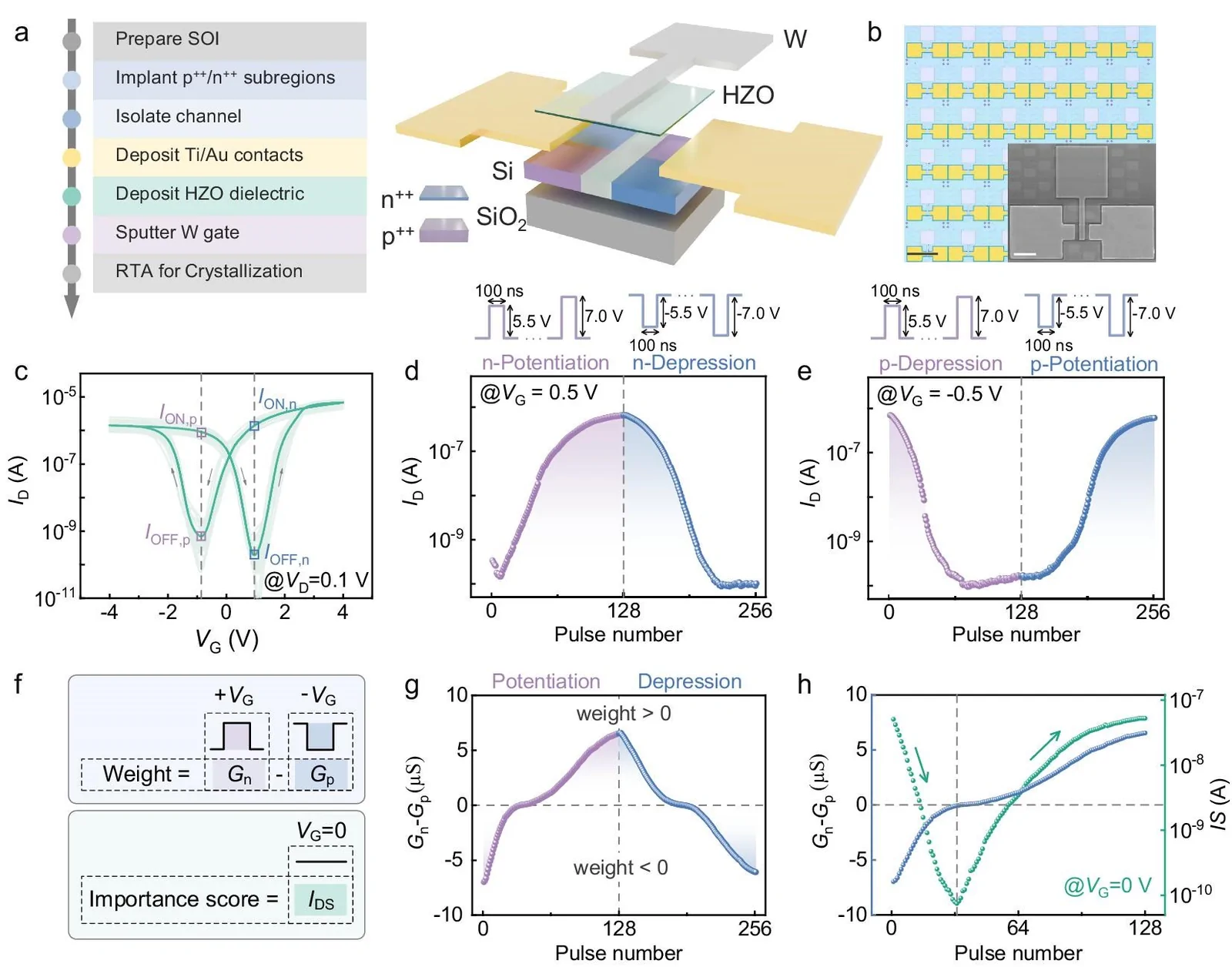
Reconfigurable weights and importance evaluation in the Fe-AmFETs. (a) Schematic device structure and fabrication flow of the Fe-AmFETs. (b) Optical microscopy image of a device array. Scale bar, 200 μm. Inset: SEM image of a typical device. Scale bar, 40 μm. (c) Transfer characteristic curves of 30 randomly sampled Fe-AmFETs. (d) Electron-dominated and (e) hole-dominated potentiation and depression behaviors in the Fe-AmFETs induced by positive and negative gate pulses, respectively. (f) Definition of weight and importance score. (g) Weight potentiation and depression behaviors. (h) Correlation between the weight values and importance scores.

The ambipolar ohmic contact and ferroelectric HZO dielectric endow the Fe-AmFETs with ambipolar transfer characteristics and nonvolatile ferroelectric memory behaviors [30,31]. As shown in the transfer characteristic (*I*_D_–*V*_G_) curves in Fig. 2c, both positive and negative gate voltages (*V*_G_) can activate the device conduction, yielding on/off ratios of nearly 10^5^ in the electron-dominated branch (*n*-mode, *I*_ON_,*_n_*/*I*_OFF_,*_n_*) and above 10^3^ in the hole-dominated branch (*p*-mode, *I*_ON_,*_p_*/*I*_OFF_,*_p_*). The former arises from electron accumulation within the Si channel, whereas the latter is attributed to hole accumulation. Moreover, the memory window (MW) expands as the sweep voltage range increases (Fig. S9a). Benefitting from mature Si technology, the result in Fig. 2c indicates that the Fe-AmFETs exhibit excellent electrical uniformity across devices, with an average MW of ∼1.78 V under a maximum sweep voltage of 4 V (Fig. S9b). Further studies demonstrate that the devices feature ultrafast programming/erasing speeds of 15/20 ns (Fig. S9c), favorable retention performance over 10^5^ s and stable endurance across 10^5^ programming/erasing cycles (Fig. S9d).

The ambipolar modulation and nonvolatile memory characteristics of the Fe-AmFETs allow the devices to perform reconfigurable long-term potentiation (LTP) and long-term depression (LTD) functions. Figure 2d depicts the LTP and LTD behaviors of a typical device working in the *n*-mode, where the output current is measured at *V*_G_ = 0.5 V to maintain electron-dominated conduction. When 128 positive pulses with linearly ramped amplitudes from 5.5 to 7.0 V are applied, the electron density within the Si channel rises continuously. This gradually boosts the output current and yields a typical LTP characteristic. In contrast, applying 128 negative pulses with amplitudes progressively increased from −5.5 to −7.0 V gradually depletes the accumulated electrons and reduces the output current accordingly, which demonstrates a typical LTD characteristic. Similarly, Fig. 2e displays the hole-dominated output current measured at *V*_G_ = −0.5 V. A sequence of 128 positive pulses whose amplitudes increase linearly from 5.5 to 7.0 V gradually reduces the accumulated holes, suppressing the output current and yielding an obvious LTD characteristic. Conversely, application of 128 negative pulses with amplitudes ranging from −5.5 to −7.0 V increases the hole density, causing a gradual rise in output current and thereby endowing the device with the LTP characteristic.

The unique reconfigurable LTP and LTD characteristics of the Fe-AmFETs allow a single device to implement both positive and negative weights. In our scheme, the weight is defined as the differential conductance (*G* = *G_n_* − *G_p_*) of the devices, where *G_n_* and *G_p_* denote the device conductance under n-mode and p-mode, respectively (Fig. 2f). In this way, continuous positive and negative weight values can be readily obtained, as shown in the potentiation and depression curves of Fig. 2g. Specifically, taking the left potentiation region as an example, a specific positive gate pulse is applied first, followed by sequential acquisition of the drain current at *V*_G_ = 0.5 V (corresponding to the current in the left region of Fig. 2d) and *V*_G_ = −0.5 V (corresponding to the current in the left region of Fig. 2e), respectively. The difference between these two currents yields an equivalent differential conductance that serves as the synaptic weight. As the two drain currents display opposite modulation trends under pulse excitation, the proposed scheme enables continuous weight tuning across negative to positive ranges. The right depression region follows exactly the same working principle. Moreover, the output characteristic *I*_D_–*V*_D_ curves in Fig. S10 show that the Fe-AmFETs can achieve 128 well-resolved output states, corresponding to an effective 7-bit weight resolution.

The ambipolar nature of the Fe-AmFETs also enables the evaluation of weight importance by defining the output current at *V*_G_ = 0 V as the importance score (IS) (Fig. 2f). This current is determined by the density of majority carriers (electrons or holes) in the channel, which is dynamically modulated by the gate pulses used for weight tuning. For instance, if one Fe-AmFET is initially configured to a *p*-type state via negative gate pulses, subsequent application of positive pulses for weight tuning gradually depletes the accumulated holes in the channel, leading to a monotonic decrease in the output current. Once the holes are fully depleted, continuous positive pulsing induces electron accumulation, causing the current to progressively rise again. Figure 2h plots the experimentally measured current at *V*_G_ = 0 V as a function of pulse number, which clearly confirms the above-discussed current-tunable characteristics of the Fe-AmFETs. Importantly, the modulation trend of this current is consistent with the absolute value of the synaptic weight. This indicates that the output current at *V*_G_ = 0 V can serve as a reliable metric for evaluating the importance of weights. Specifically, a larger absolute weight value corresponds to a higher importance level, which is manifested as a larger output current at *V*_G_ = 0 V and vice versa. Discussions regarding the definition of the differential conductance as the weight and the output current at *V*_G_ = 0 V as the IS are detailed in Note S1.

### Adaptive pruning convolutional neural network based on the Fe-AmFET crossbar arrays

Leveraging the inherent weight tuning and importance evaluation capabilities, we develop an APCNN based on Si Fe-AmFET crossbar arrays. As shown in Fig. 3a, the network consists of two convolutional layers (Conv1 and Conv2), two max-pooling layers, one fully connected layer and one output layer. Each convolutional layer is implemented by an independent Fe-AmFET crossbar array. Figure 3b illustrates the schematic of the crossbar arrays (size: *M* × *N*) and their peripheral modules, where the word lines (WLs), bit lines (BLs) and source lines (SLs) are connected to the gate, drain, and source terminals of the Fe-AmFETs, respectively. A column-wise strategy is adopted to map convolutional operations onto each array [17,32,33]. Specifically, for the first crossbar array (Conv1, *M* = 3 × 3, *N* = 32), the weights of each convolutional kernel (size: 1 × 3 × 3) are unrolled and mapped to a dedicated column of the array composed of 3 × 3 Fe-AmFETs. For the second crossbar array (Conv2, *M* = 32 × 3 × 3, *N* = 64), the weights of each convolutional kernel (size: 32 × 3 × 3) are similarly unrolled and mapped to a specific column of the array. Consequently, the *N* columns of each crossbar array can simultaneously execute *N* convolutional kernels in parallel. That is to say, the first crossbar array implements 32 convolutional kernels for Conv1, and the second performs 64 convolutional kernels for Conv2. For weight updating, programming pulses are applied to the WLs of each crossbar array, while column selection is achieved by biasing the corresponding SLs with specific voltage levels. This design enables independent tuning of the weights of each Fe-AmFET in Conv1 and Conv2 (Fig. S11).

**Figure 3.**
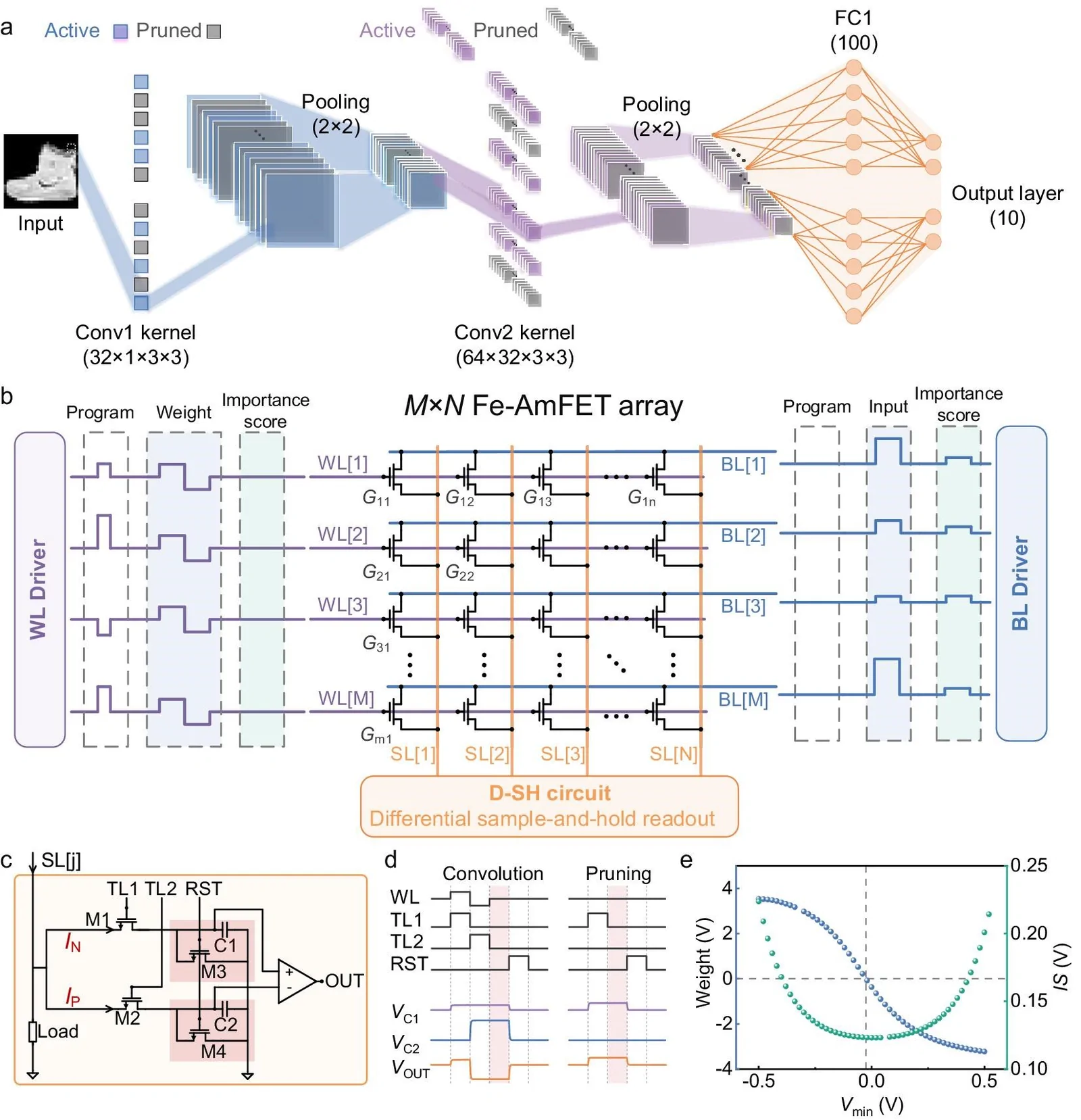
Adaptive pruning network based on the Fe-AmFET crossbar arrays. (a) Illustration of the network architecture. (b) Schematic of the Fe-AmFET crossbar array and its peripheral driving circuitry. (c) Differential sample-and-hold (D-SH) readout circuit and (d) its corresponding timing signals. (e) SPICE-simulated correlation of the readout importance scores and weight values.

For convolutional operations, input values are mapped to different voltage levels and applied to the BLs. Currents are initially sampled from the SLs when a positive read voltage is applied to the WLs, configuring the Fe-AmFETs in *n*-mode. Current sampling is then performed under a negative read voltage to operate the Fe-AmFETs in *p*-mode. The differential output current at the *j*-th SL (*I_j_*) can be derived via the following equation:


(1) \begin{eqnarray*}
{I}_j &=& \mathop \sum \limits_{i = 1}^m \left( {{G}_{ij,n}{V}_i - {G}_{ij,p}{V}_i} \right)\\
&=& \mathop \sum \limits_{i = 1}^m \left( {{G}_{ij,n} - {G}_{ij,p}} \right){V}_i = \mathop \sum \limits_{i = 1}^m {G}_{ij}{V}_i,
\end{eqnarray*}

where *V_i_* is the input voltage at the *i*th BL, *G_ij,n_* and *G_ij,p_* are the conductances of the Fe-AmFET at the *i*th row and *j*th column working in *n*-mode and *p*-mode, respectively, and *G_ij_* is the corresponding differential conductance. Following this principle, the crossbar arrays can physically execute MAC operations between input values and stored weights, enabling *in situ* hardware implementation of convolutional processes.

For importance evaluation, all WLs are grounded, and a fixed read-enable voltage (*V*_read_) is applied to the BLs. The importance current (*I*_im,*j*_) collected from the *j*th SL, which corresponds to the *j*th kernel, follows the equation:


(2) \begin{eqnarray*}
{I}_{{\mathrm{im}},j} = \mathop \sum \limits_{i = 1}^m {I}_{{\mathrm{im}},ij},
\end{eqnarray*}

where *I*_im_,*_ij_* denotes the current of the Fe-AmFET at the *i*th row and *j*th column under zero gate biases.

In addition, a custom differential sampling-hold (D-SH) readout circuit is designed to perform current-difference operations in the analog domain, with each kernel column paired with an independent D-SH readout circuit to collect feature signals. Figure 3c and d depicts the D-SH circuit and its corresponding timing control signals, respectively. For convolutional operations, the Fe-AmFETs first operate in *n*-mode, turning on transistor M1 and allowing current to charge capacitor C1. The devices then switch to *p*-mode, with transistor M2 turning on to charge capacitor C2. Owing to the retention property of the sampling capacitors, the D-SH circuit can perform current subtraction for the SL outputs. For the importance current readout, only transistor M1 is turned on since the difference operation is not required. To reset the circuit, transistors M3 and M4 are turned on after each operation cycle to discharge the residual charges on capacitors C1 and C2. The results simulated using the simulation program with integrated circuit emphasis (SPICE) in Fig. 3e demonstrate that the circuit can effectively convert the SL currents into differential outputs, and the importance current outputs accurately reflect the corresponding weight magnitudes, as detailed in Fig. S12.

### Performance evaluation of the adaptive pruning convolutional neural network

Figure 4a demonstrates the workflow of the APCNN. Following network pre-training, all convolutional kernels are ranked based on the output importance currents (i.e. IS) collected from the Fe-AmFET crossbar arrays. A pruning threshold current is then determined based on this ranking and the expected pruning ratio for each round. Kernel columns with importance currents below the threshold are identified as redundant kernels and electrically disconnected from their corresponding D-SH readout circuits. Meanwhile, the corresponding D-SH readout circuits are synchronously power-gated to eliminate unnecessary power overhead. After that, the network with remaining kernels is fine-tuned to compensate for sparsity to optimize the accuracy. This pruning-and-fine-tuning cycle is iterated repeatedly until the network meets the expected accuracy requirement for specific tasks, as detailed in Note S2.

**Figure 4.**
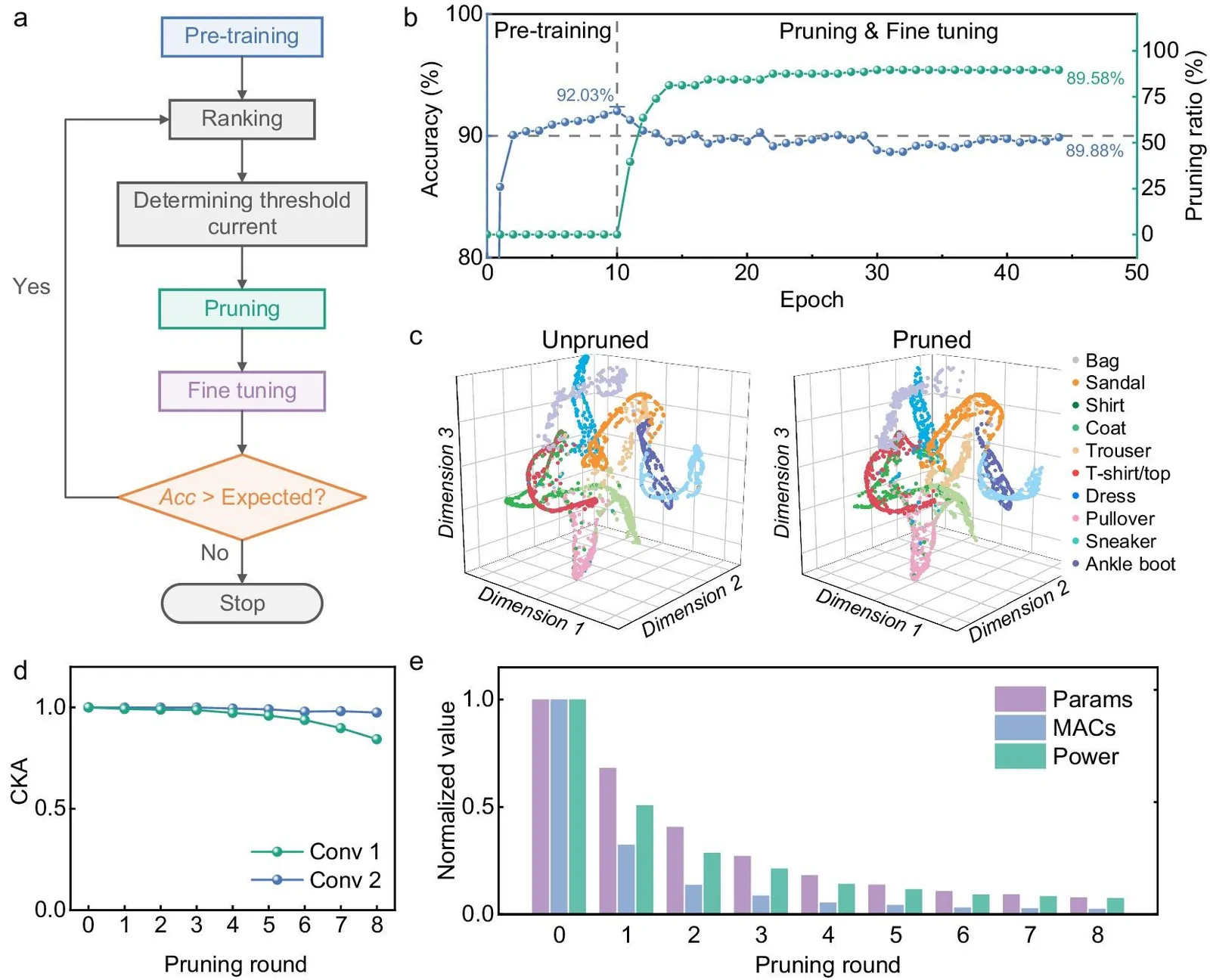
Performance evaluation of the adaptive pruning convolutional neural network. (a) Schematic of the pruning workflow. (b) Relationship between the inference accuracy and pruning ratio of the hardware network. (c) *t*-SNE visualization of the feature manifolds. (d) CKA analysis of feature fidelity across convolutional layers (Conv1 and Conv2). (e) Pruning benefits of the network. All evaluations are performed on the Fashion-MNIST dataset.

To validate the feasibility, the network is evaluated on the Fashion-MNIST dataset. As shown in Fig. 4b, the number of convolutional kernels is drastically reduced after eight pruning rounds, yielding a kernel pruning ratio of 89.58%, while the inference accuracy only slightly decreases from 92.03% to 89.88%. This verifies that the Si Fe-AmFET crossbar array APCNN can effectively eliminate redundant kernels while preserving high inference accuracy. Detailed configurations of the convolutional kernels before and after pruning are provided in Fig. S13. For practical deployment, the hardware inevitably suffers from non-idealities like device-to-device variation, temporal fluctuation and programming noise. To validate the practical reliability of our scheme, we incorporate Gaussian perturbations to simulate such static and dynamic noises and study their influence on kernel pruning decisions and overall network performance. As shown in Note S3, the results indicate that the Fe-AmFET-based adaptive pruning scheme exhibits favorable robustness against these hardware non-idealities.

To validate that the pruning strategy does not degrade the network inference capability, *t*-distributed stochastic neighbor embedding (*t*-SNE) is adopted to visualize the high-dimensional feature maps extracted by the APCNN [34,35]. As a powerful dimensionality reduction tool, *t*-SNE projects high-dimensional data into a low-dimensional space, where data points with similar feature distributions cluster closely together. As shown in Fig. 4c, the feature manifolds of the pruned network form well-separated clusters, which are highly consistent with those of the original unpruned network. This demonstrates that the network’s feature representation capability remains robust following eight rounds of structural pruning. Even with a heavily pruned architecture, the network still effectively retains the core classification features of the original network.

Linear centered kernel alignment (CKA) is also used to quantitatively evaluate the feature space fidelity between the pruned and original networks [36]. The results in Fig. 4d show that Conv1 achieves a high CKA fidelity score of 0.975, demonstrating that low-level visual features are well preserved even after the drastic reduction in the kernel count. For Conv2, the CKA score decreases to 0.843, which indicates that our pruning-and-fine-tuning strategy enables the network to adapt its high-level feature extraction to pruning-induced structural modifications, while retaining the core feature information critical for classification inference.

To quantify the benefits of the APCNN, a systematic evaluation is conducted in terms of parameter count (Params), MAC operations and inference power consumption (see Note S2 for details). Specifically, the parameter count indicates the memory overhead of the network, while the MAC operations serve as a metric for quantifying its computational complexity. As shown in Fig. 4e, our hardware-based pruning strategy achieves a substantial reduction in overall resource consumption. The total parameter count is reduced by 92.2% from 334 168 to 26 188, and MAC operations drop by 97.5% from 4153 064 to 104 880. Notably, the structural sparsity induced by pruning enables the Si Fe-AmFET crossbar array to reduce its intrinsic power consumption by 98.7%, dropping from 428.76 to 5.64 μW. When incorporating the power consumption of peripheral D-SH readout circuits, the overall core inference power consumption is reduced by 92.4%, decreasing from 1394.52 to 106.24 μW. Such significant energy efficiency gains are essential for the practical deployment of neuromorphic networks in energy-constrained edge computing scenarios [37,38].

In addition, to comprehensively assess the Fe-AmFET-based APCNN, we compare its training capability and pruning efficacy against multiple baselines across the Fashion-MNIST, MNIST and CIFAR-10 datasets. As shown in Fig. 5a–c, the hardware-implemented APCNN achieves test accuracies and training convergence behaviors comparable to those of its full-precision software baselines. For the pruning efficacy, random pruning (averaged over 10 independent trials) and conventional software-based L1-Norm pruning strategies are employed for the comparison [39,40]. Notably, no fine-tuning is performed for any of the strategies after pruning to better demonstrate the inherent pruning capability. The results in Fig. 5d–f indicate that the Fe-AmFET-based APCNN delivers comparable performance to the L1-Norm pruning but outperforms the random pruning. Moreover, the superiority over the random pruning is more prominent as the dataset complexity increases. For instance, at the pruning ratio of 30%, the Fe-AmFET-based APCNN incurs a marginal relative accuracy drop of 1.06% (from 98.94% to 97.89%) on the simple MNIST dataset, which is comparable to the 0.80% loss of the L1-Norm pruning but slightly lower than the 1.61% accuracy degradation caused by the random pruning. For the most complex CIFAR-10 dataset, the Fe-AmFET-based APCNN exhibits a relative accuracy decline of 25.72% (from 72.00% to 53.48%), while the L1-Norm and random pruning result in accuracy losses of 26.36% and 41.72%, respectively. Additionally, taking the Fashion-MNIST results in Fig. 5d as an example, the accuracy remains nearly invariant at pruning ratios below 25%, while accuracy degrades evidently once the pruning ratio exceeds 30%, indicating further compression will remove functionally critical weights. For aggressive scenarios without post-pruning fine-tuning, a trade-off between classification accuracy and network sparsity (pruning ratio) is essential.

**Figure 5.**
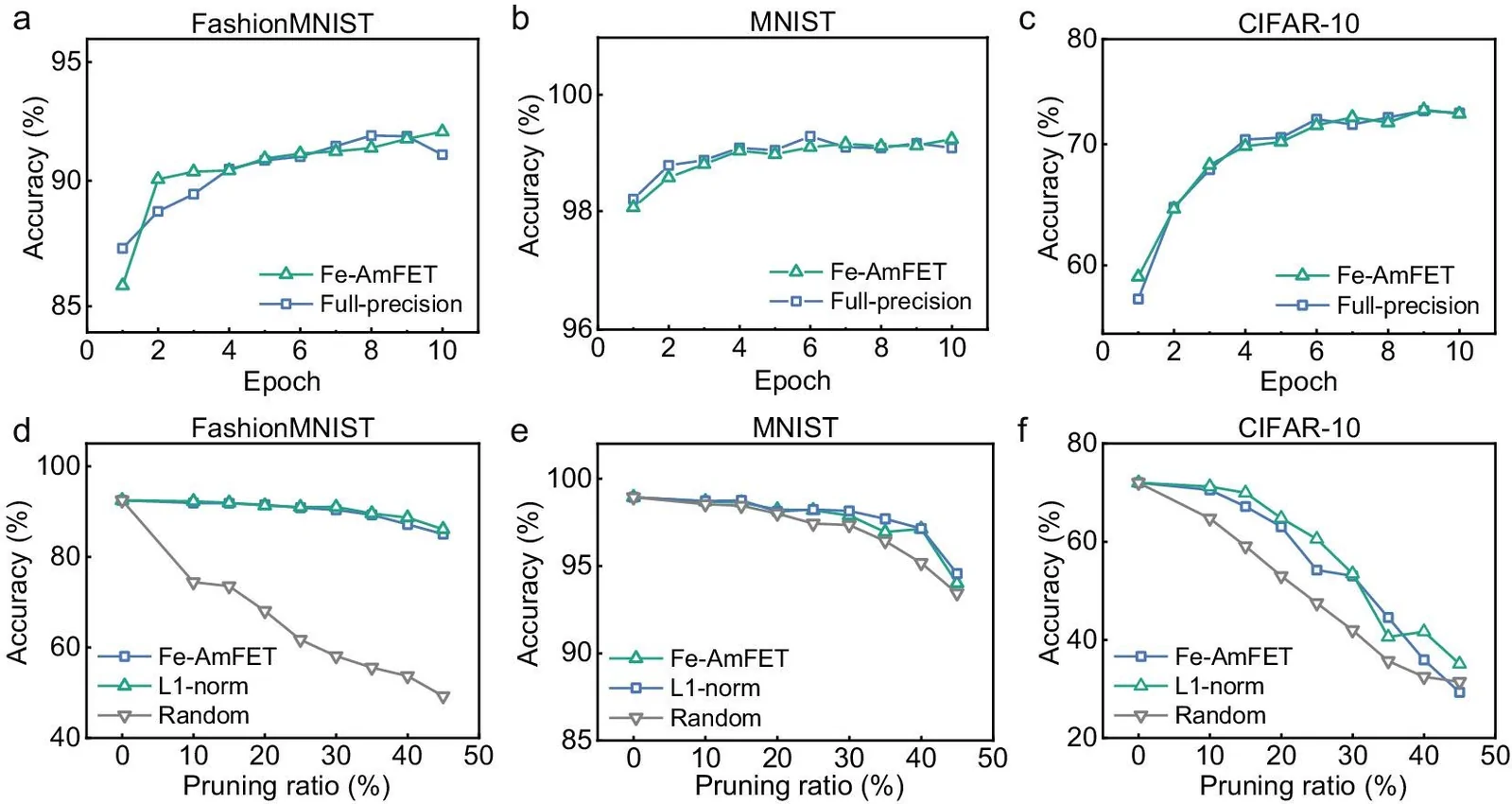
Benchmarking of training and pruning performance across different datasets. Test accuracy comparison between the Fe-AmFET-based network and full-precision (FP32) software baselines on the (a) Fashion-MNIST, (b) MNIST and (c) CIFAR-10 datasets. Pruning efficacy of different strategies on the (d) Fashion-MNIST, (e) MNIST and (f) CIFAR-10.

## DISCUSSION

In summary, this work demonstrates a hardware-efficient, brain-inspired APCNN based on the Si Fe-AmFET crossbar arrays. The fabricated Si Fe-AmFETs exhibit robust ferroelectric memory characteristics and reconfigurable ambipolar transport behaviors, allowing us to implement both positive and negative synaptic weights within a single device and evaluate synaptic importance via their zero-gate-voltage output currents. Leveraging these intrinsic device properties, we construct a hardware network based on Si Fe-AmFET crossbar arrays that integrates MAC operations, nonvolatile weight storage and *in situ* kernel pruning. Our studies indicate that the hardware implementations deliver extraordinary gains in kernel pruning ratio, network parameter compression, MAC operation reduction and power savings, with performance comparable to that of traditional software pruning approaches across various benchmark datasets. The scalability of the hardware architecture is also validated via ResNet on the CIFAR-10 dataset (see Note S4 for details). For practical deployment, the endurance of our current devices may not be sufficient to support the massive weight update operations required for full *in situ* training. Nevertheless, FeFET-based synaptic devices have shown great potential for IMC and on-chip training [41], with optimized devices achieving endurance over 10^10^ program/erase cycles [42]. Therefore, properly optimized Fe-AmFETs are expected to meet the endurance requirement for full *in situ* training and are promising for adaptive pruning network hardware.

## METHODS

### Device fabrication

The Fe-AmFETs were fabricated on an SOI wafer featuring a 100-nm-thick device layer with a resistivity exceeding 1000 Ω·cm. The detailed fabrication flow is schematically demonstrated in Fig. S6. First, photolithography and ion implantation were adopted to introduce phosphorus and boron dopants into the device layer to form source and drain terminals. Each source/drain contains one *n*^++^ and one *p*^++^ subregion, both with a doping concentration of ∼10^20^ cm^−3^. Second, device isolation was implemented. The device layer was patterned via photolithography and reactive ion etching to isolate discrete device mesas, and the etched trenches were filled with Al_2_O_3_ film. Third, Ti/Au stacks were deposited on the source and drain by electron beam evaporation, after which rapid thermal processing (RTP) was carried out to achieve ambipolar ohmic contacts. Fourth, a ferroelectric HZO film was grown by atomic layer deposition, followed by the deposition and patterning of tungsten (W) as the top-gate electrode. Finally, RTP was conducted at 500°C for 30 s in a nitrogen atmosphere to crystallize HZO to form the ferroelectric phase.

### Characterizations

All electrical characterizations were carried out at room temperature in ambient air using a probe station equipped with a semiconductor parameter analyzer.

### Simulations

Device simulations of the Fe-AmFETs were performed using technology computer-aided design (TCAD), with device physics described by coupled Poisson and continuity equations. The simulation setup incorporates the Shockley–Read–Hall recombination model alongside sophisticated mobility models, including the Philips Unified Mobility (PhuMob) model, the Enormal model accounting for vertical-field-induced mobility degradation and the High-Field Saturation model to capture velocity saturation that is critical for evaluating short-channel devices.

Circuit simulations were performed using LTspice. The simulations targeted the critical readout path of the crossbar array, with the incorporation of the custom D-SH circuit. To emulate the worst-case operating conditions in large-scale arrays, parasitic interconnect resistance (*R*_par_) and bit-line capacitance (*C*_par_) are explicitly incorporated into the netlist. Detailed simulation parameters and circuit configurations are summarized in Table S5 and Fig. S12.

## Supplementary Material

nwag547_Supplemental_File

## Data Availability

The data that support the findings of this study are available from the corresponding author upon reasonable request.
