# Supplementary material for: A hardware-implemented adaptive pruning neural network
Source: Natl Sci Rev. 2026 Aug 27;13(20):nwag547. doi: 10.1093/nsr/nwag547 (PMC13729758; doi:10.1093/nsr/nwag547)
Supplement: Supplementary file 1 [file nwag547_supplemental_file.pdf]

Supplementary Information

A hardware-implemented adaptive pruning neural network

Yusen Tian, Penghao Chen, Ziyu Ming, Zengxing Zhang*

School of Microelectronics, Fudan University, Shanghai 200433, China

*E-mail: zhangzx@fudan.edu.cn

Note S1. Theory for defining the differential conductance as the weight and the output current at V_G=0 V as the importance score.

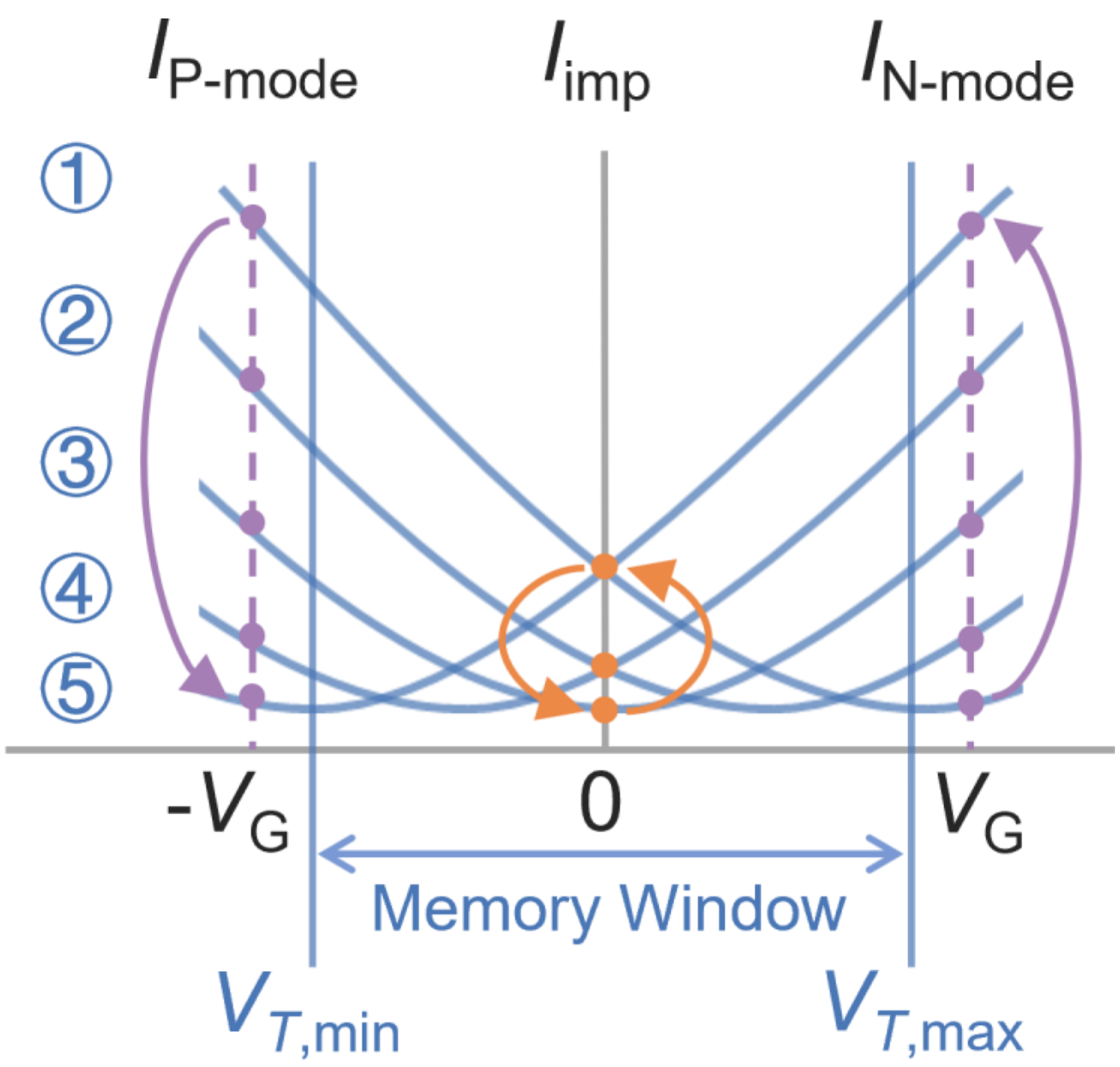

Figure S1. Schematic illustration of the Fe-AmFET transfer characteristic curves under various programmed memory states, alongside the read gate voltages $\pm V_G$ adopted to acquire differential conductance.

Figure S1 schematically illustrates the working principle for achieving synaptic weight (differential conductance) and importance score (connection importance current) in the Fe-AmFET. Curves 1-5 display the transfer characteristic curves of the Fe-AmFET programmed into five distinct memory states via positive gate pulses, where $\pm V_G$ denote the read gate voltages. Notably, the absolute value of the adopted $\pm V_G$ is larger than the absolute gate voltage corresponding to the valley point of every

ambipolar transfer curve, which guarantees the device operate in the electron-dominated and hole-dominated conduction regimes, respectively.

For the Fe-AmFET, the threshold voltages of the electron branch (V_{Tn}) and hole branch (V_{Tp}) follow classical metal-oxide-semiconductor FET (MOSFET) physical models,

$$V_{Tn} = V_{FB} + 2\psi_B + \frac{\sqrt{2q\varepsilon_s N_B 2\psi_B}}{C_{OX}}, \quad (\mathrm{S}-1)$$

$$V_{Tp} = V_{FB} - 2\psi_B + \frac{\sqrt{2q\varepsilon_s N_B 2\psi_B}}{C_{OX}}, \quad (\mathrm{S}-2)$$

$$\psi_B = \frac{E_F - E_i}{q}, \quad (\mathrm{S}-3)$$

where V_{FB} is the flat-band voltage, ψ_B is the Fermi potential, q is the elementary charge, ε_s is the Si permittivity, N_B is the doping concentration, C_{OX} is the gate oxide capacitance per unit area, E_F is the Fermi level, and E_i is the intrinsic Fermi level.

The Si in the device is nearly intrinsic with an extremely low doping concentration. That is to say, N_B is negligible and ψ_B is approximately 0. Accordingly,

$$V_T \approx V_{Tn} \approx V_{Tp}\,. \quad (\mathrm{S}-4)$$

Here, V_T corresponds to the voltage at the bottom of the V-shaped curves.

For a gate bias $V_G>V_T$, the hole current is suppressed and the electron current becomes dominant. Assuming the device operates in the linear region, the drain current I_D,

$$I_D = \mu_n C_{OX}\frac{W}{L}\left[(V_G - V_T)V_D - \frac{{V_D}^2}{2}\right]. \quad (\mathrm{S}-5)$$

In contrast, when $V_G<V_T$, the electron current is suppressed and the hole current dominates. The linear-region drain current is given by

$$I_D = \mu_p C_{OX}\frac{W}{L}\left[(V_T - V_G)V_D - \frac{{V_D}^2}{2}\right]. \quad (\mathrm{S}-6)$$

In our work, the synaptic weight is the differential conductance between the electron and hole branches at the corresponding read gate biases of $\pm V_G$. At a fixed drain voltage V_D, the current I_{weight} corresponding to a specific weight is,

$$I_{weight} = I_D(V_G) - I_D(-V_G). \qquad (S-7)$$

Defining $K_n = \frac{1}{2}\mu_n C_{OX}\frac{W}{L}$ and $K_p = \frac{1}{2}\mu_p C_{OX}\frac{W}{L}$ as the transconductance parameters for electrons and holes, respectively, then

$$I_{weight} = 2V_D\left[(K_n - K_p)V_G - (K_n + K_p)V_T\right] + (K_p - K_n){V_D}^2. \quad (S-8)$$

Under small V_D (strict linear-region operation), S-8 can be simplified as,

$$I_{weight} = 2V_D\left[(K_n - K_p)V_G - (K_n + K_p)V_T\right]. \qquad (S-9)$$

Then the differential conductance corresponding to the synaptic weight is,

$$weight = 2(K_n - K_p)V_G - 2(K_n + K_p)V_T. \quad (S-10)$$

Here the first term $2(K_n - K_p)V_G$ is a constant offset, which originates from the intrinsic mobility difference between the electrons and holes in Si. In our hardware-implemented neural networks, this offset can be automatically compensated by the bias parameters of the subsequent neuron layer throughout the training process. As such, the difference of the ON/OFF current ratios in the n-type and p-type branches, which mainly originates from the mobility difference, exerts negligible influence on the overall functional performance of the neural networks.

To evaluate the connection importance, the read gate voltage is set to zero (V_G=0 V), at which the devices operate near the bottom of the V-shaped curves, i.e. the subthreshold regime. In this case, the current (I_{subth}) is no longer dominated by a single type of carriers, but by the sum of subthreshold currents of electrons (I_n) and holes (I_p),

$$I_{subth} = I_n + I_p. \qquad (S-11)$$

According to the subthreshold transport theory, for n-type,

$$I_D = I_{spec}\, exp\left(\frac{V_G - V_T}{n_n\phi_t}\right)\left[1 - exp\left(-\frac{V_D}{\phi_t}\right)\right], \quad (S-12)$$

for p-type,

$$I_D = I_{spec}\, exp\left(\frac{V_T - V_G}{n_p\phi_t}\right)\left[1 - exp\left(-\frac{V_D}{\phi_t}\right)\right], \quad (S-13)$$

where I_{spec} is the specific current, n_n and n_p are the subthreshold swing factors, and

$\phi_t = \frac{kT}{q}$ is the thermal voltage. Since V_D is fixed during the importance current read-out, the V_D-dependent term can be incorporated into a factor I_0. V_G=0 and assuming symmetric subthreshold slopes for electrons and holes ($n_n \approx n_p \approx n$), the connection importance current I_{imp} is:

$$I_{imp} \approx I_0 \left[exp\left(-\frac{V_T}{n\phi_t}\right) + exp\left(\frac{V_T}{n\phi_t}\right)\right] \propto cosh(V_T). \quad (S-14)$$

Considering Equation S-10,

$$I_{imp} \propto cosh(weight). \quad (S-15)$$

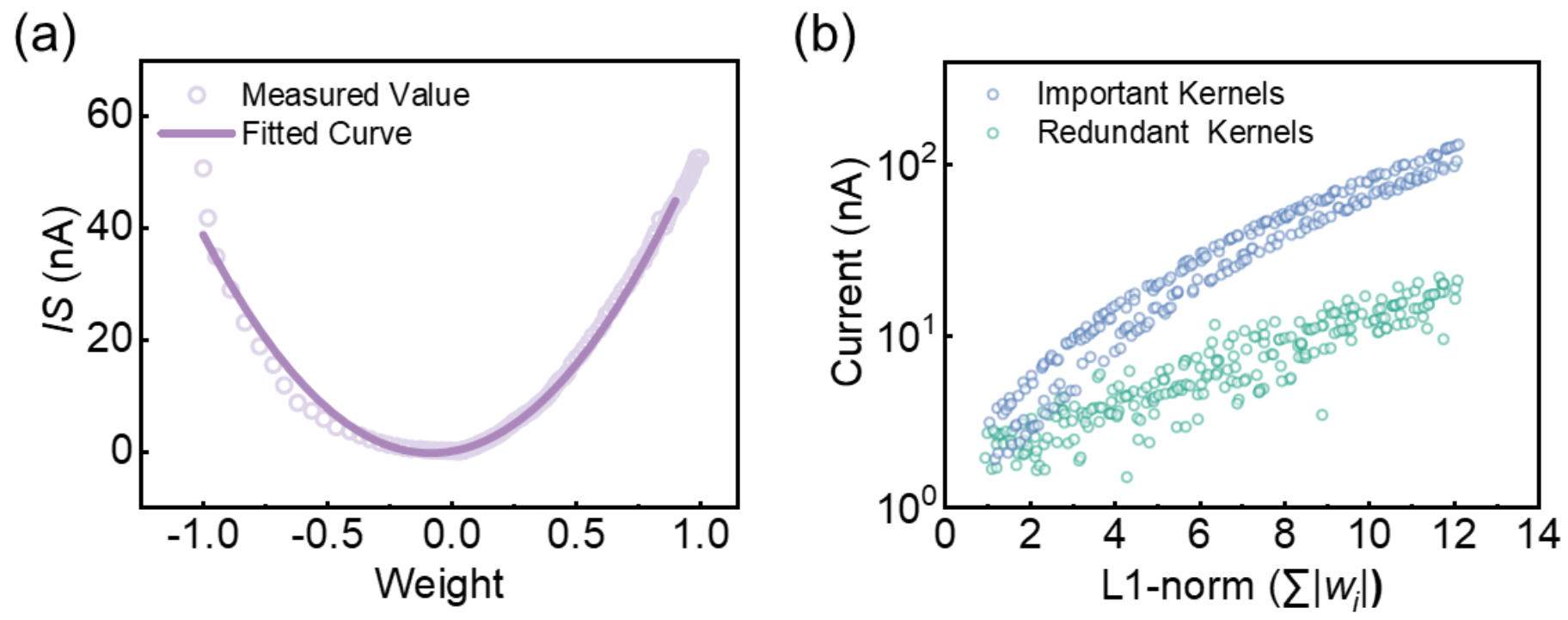

Figure S2. Quantitative relationship between the weight and importance score. (a) Experimentally acquired importance score (*IS*) plotted against weight value, which can be fitted well with a hyperbolic-cosine function. (b) Simulated importance current distribution for 500 pairs of important and redundant 3×3 kernels. Each pair shares the same L1-norm but differs in internal weight distributions.

Equation S-15 indicates that the connection importance current I_{imp}, i.e. *IS*, follows a hyperbolic cosine-like relationship with the weight value, a characteristic that delivers favorable mathematical properties for network pruning. Based on this dependence, I_{imp} reaches its minimum value at *weight*=0 and rises as the absolute weight value increases. For weights near zero (redundant small weights), the hyperbolic-cosine function exhibits a shallow slope, so weight variations only trigger tiny shifts in I_{imp}. In contrast, large-magnitude weights correspond to a steep function slope, where the same weight perturbation leads to dramatic changes in I_{imp}. This intrinsic nonlinear characteristic enables the fundamental physical mechanism to suppress redundant small weights while enhancing important large weights during

hardware-native kernel screening. To verify this relationship, we fit experimentally measured currents across all 128 discrete weight states of our Fe-AmFET to the hyperbolic cosine model. The resulting coefficient of determination reaches R^2=0.993 (Fig. S2a), confirming the robust correlation between *IS* and the weight value.

In addition, we carried out a kernel-level simulation to evaluate the feasibility of the above-discussed principle. It is performed on 500 pairs of important and redundant 3×3 kernels. For each pair, the two kernels share a same L1-norm value,

$$\sum_i |weight_i^{red}| = \sum_i |weight_i^{imp}|, \qquad (S-16)$$

where $weight_i^{red}$ and $weight_i^{imp}$ refer to weights inside redundant and important kernels, respectively. The redundant kernels are constructed with uniformly distributed low-magnitude weights, simulating smooth background noise and non-discriminative redundant features. The important kernel has the same L1-norm, but its weight magnitude is concentrated in only one or two synapses, while the remaining weights are close to zero. This distribution mimics sparse but salient local features, such as edges or key texture components. As demonstrated in Fig. S2b, despite having same software L1-norms, important kernels generate a higher current than redundant kernels, indicating that redundant small weights are suppressed while important weights are enhanced. In contrast, such a feature is difficult to be achieved in conventional software-based pruning, where L1-norm ($\sum |weight_i|$) is employed as the standard metric to quantify kernel importance. This linear scoring scheme fails to distinguish weight distributions. A dense kernel populated with numerous small redundant weights can receive an identical importance score to a sparse kernel holding only one or two large informative weights.

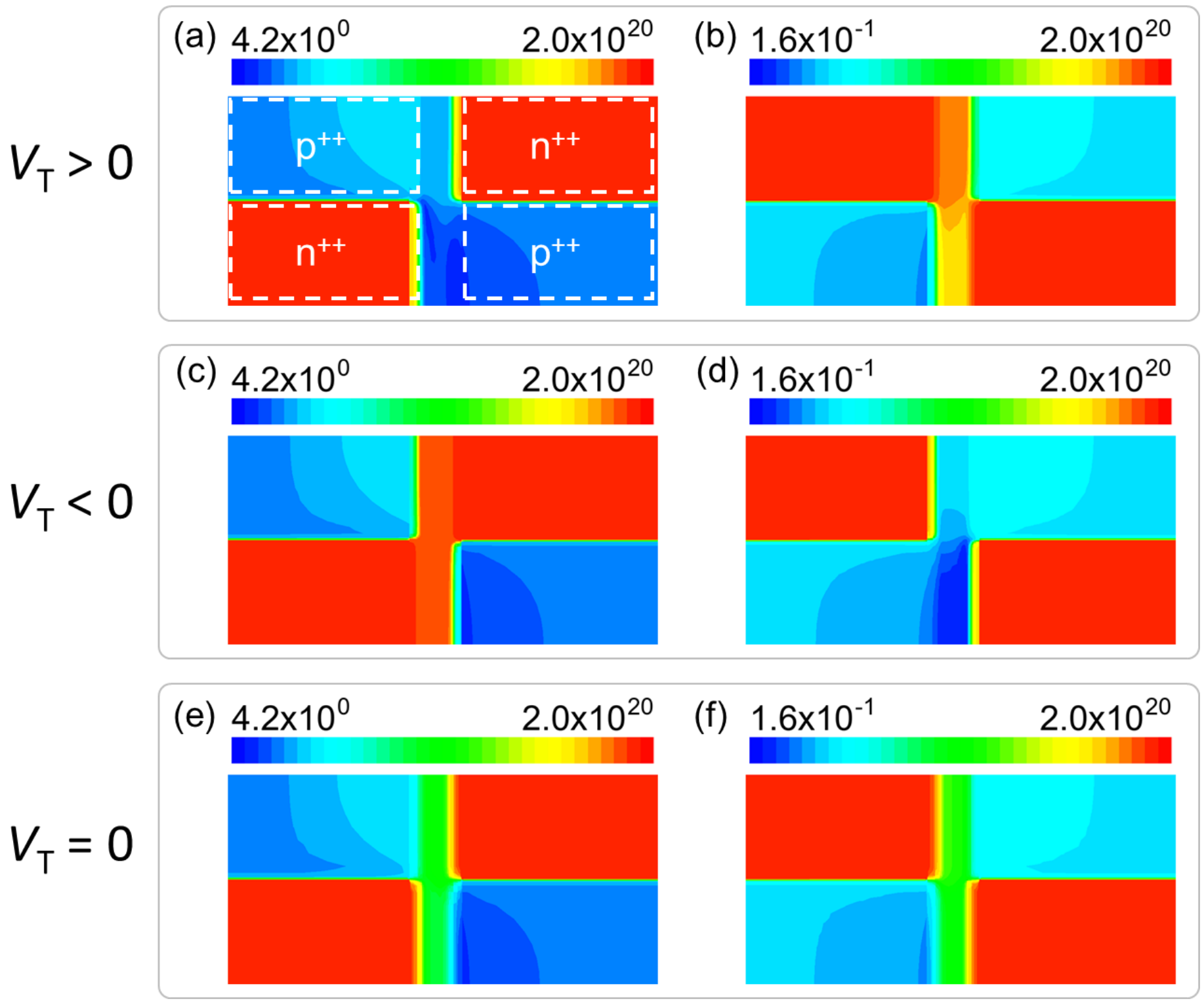

Figure S3. TCAD-simulated carrier distributions in the channel of the Fe-AmFET under different ferroelectric polarization states and the V_G=0 V read condition. (a) and (b) V_T>0 for electrons and holes, respectively. (c) and (d) V_T<0 for electrons and holes, respectively. (e) and (f) V_T=0 for electrons and holes, respectively.

To further validate the operating principle, we conducted TCAD simulations to visualize the carrier distributions in the channel under different ferroelectric polarization states and the V_G=0 V read condition. For V_T>0 (Fig. S3a and Fig. S3b), the ferroelectric polarization vector aligns toward the W gate electrode layer, rendering the channel hole-dominated with a high hole concentration. Conversely, for V_T<0 (Fig. S3c and Fig. S3d), the ferroelectric polarization vector aligns toward the Si channel, and the channel becomes electron-dominated with abundant electrons. At the nearly neutral state V_T=0, the ferroelectric polarization is negligible, so both electron and hole densities remain extremely low.

Note S2. Definition and calculation of performance metrics.

1. Pruning ratio definition and detailed pruning strategy.

Three pruning ratios are defined for the neural network pruning: the per-round pruning ratio r_t, the cumulative pruning ratio R_t, and the final overall pruning ratio. Specifically, r_t denotes the fraction of kernels removed in the t-th pruning round relative to the total number of kernels in the initial network; R_t represents the cumulative fraction of kernels pruned from the initial network after t pruning rounds; the final overall pruning ratio corresponds to the total fraction of kernels removed upon termination of the pruning procedure.

In our work, we adopt a progressive pruning strategy. At early pruning rounds, the network has abundant redundant kernels, allowing us to prune a larger fraction of kernels per round, i.e., a larger r_t. As pruning progresses, the network becomes increasingly vulnerable to performance degradation, so we decrease r_t accordingly. After the per-round pruning ratio r_t is specified, the threshold is derived from the ranked importance scores. Kernels with the lower scores are then pruned, followed by fine-tuning and accuracy evaluation. The iterative pruning process runs continuously until the accuracy loss falls within the allowed tolerance. Notably, the pruning ratios adopted in our current implementation are empirically specified scheduling hyperparameters for each task. This strategy is widely used in conventional filter pruning studies [1-3]. To enable fully adaptive hardware deployment, an automatic feedback-driven pipeline should be developed in future work.

In addition, for hardware implementation, kernels are mapped to independent columns of the Fe-AmFET crossbar array via a column-wise mapping scheme, where each kernel column is paired with an independent D-SH readout circuit for feature signal collection. The importance current collected from each Fe-AmFET column is compared with the threshold determined by the task-specific target pruning ratio to screen out redundant kernels. Once a kernel column is identified as redundant, it will be marked as inactive, and its connection to the corresponding readout circuit will be electrically disconnected by peripheral control circuits. All non-redundant kernel

columns maintain conductive readout channels to enable current collection and MAC computation. Notably, our design achieves dual power reduction. In addition to cutting off the crossbar array conduction paths of redundant kernels, the matched D-SH readout circuits for all inactive kernel columns are synchronously disabled to eliminate their power overhead. This co-shutdown of array access paths and peripheral readout modules will effectively suppress the total system inference power.

2. Parameter count (Params).

The total number of parameters reflects the hardware overhead of neural networks, including weights and biases of convolutional (Conv) and fully-connected (FC) layers. The overall parameter count is obtained via the equation,

$$Params = \sum_{l=1}^{L} \left(C_{out}^{l} \times C_{in}^{l} \times K_h \times K_w + C_{out}^{l} \right). \quad (S-17)$$

3. Multiply-accumulate (MAC) operation count.

The total number of MAC operations reflects the computational complexity of neural networks. The overall MAC operation count for convolutional (Conv) layers is obtained via the equation,

$$MACs = \sum_{l=1}^{L} \left(H_{out}^{l} \times W_{out}^{l} \times K_h \times K_w \times C_{out}^{l} \times C_{in}^{l} \right). \quad (S-18)$$

4. Power consumption.

We evaluate the core power consumption of inference by incorporating the crossbar array and D-SH readout circuits.

For the crossbar array power P_{array}, it accounts for the conduction power of activated Fe-AmFET devices and is calculated using experimentally characterized currents working in n-mode and p-mode, respectively. For a single device at row *i* and column *j*, the power dissipation is given by,

$$P_{ij} = \frac{V_D \times \left(I_{N,ij} + I_{P,ij} \right) \times t}{T}. \quad (S-19)$$

where the source-drain bias is fixed at $V_D = 0.1\,V$.

Then the power consumption of the arrays is,

$$P_{array} = \sum_{j=1}^{N} \sum_{i=1}^{M} P_{ij}, \qquad (S-20)$$

For the readout circuit power, we model the crossbar load in SPICE with an equivalent resistor calibrated to the measured resistance of the Fe-AmFET, ensuring the simulation faithfully reproduces real loading conditions during read operations. The average power of a single D-SH readout circuit is derived by integrating instantaneous supply power over one complete read cycle,

$$P_{D-SH} = \frac{1}{T} \int_{0}^{T} V_{DD} I_{DD}(t)\, dt. \qquad (S-21)$$

Then the core power consumption of inference, including the array and the D-SH readout circuits, is estimated as

$$P_{infer.core} = P_{array} + N_{active} P_{D-SH}, \qquad (S-22)$$

where N_{active} is the number of active kernels.

For the Fashion-MNIST dataset, our simulations indicate that the power consumption of the crossbar array drops from 428.76 μW to 5.64 μW after eight pruning rounds, representing a 98.7% reduction. The total power consumption of one D-SH readout circuit includes dynamic and static power. The dynamic power, incurred during charging and discharging of sampling capacitors, averages ~8.75 fW, with a peak transient power of ~1.84 pW. The static power, which primarily originates from the constant bias current required for implementing analog subtraction, averages ~10.06 μW per unit per read cycle. Obviously, the dynamic power is negligible compared to the static power. Consequently, the power dissipation of each D-SH readout circuit is approximated as 10.06 μW in a single read cycle. Our simulations further demonstrate that the power consumption from the readout circuits decreases from 965.76 μW to 100.60 μW after eight pruning rounds. Combining the power contributions from the crossbar array and readout circuits, the overall core power consumption of inference falls from 1394.52 μW to 106.24 μW after eight pruning rounds, corresponding to a reduction of 92.4%.

In addition, each complete differential readout consists of five 1-μs timing phases,

resulting in a total readout time of 5 μs. Therefore, the inference core energy consumption per complete vector-matrix multiplication (VMM) is reduced from $1394.52\ \mu\mathrm{W}\ \times 5\ \mu\mathrm{s} = 6.97\ \mathrm{nJ}$ to $106.24\ \mu\mathrm{W}\ \times 5\ \mu\mathrm{s} = 0.531\ \mathrm{nJ}$.

5. List of symbols used in the equations.

Table S1 shows the symbols used in the equations.

Table S1. List of symbols.

Symbol	Full Name	Description
t	Sampling Time	The time of n-mode or p-mode currents are integrated onto the sampling capacitors.
T	Readout Time	The time of a complete MAC operation.
L	Layers	The number of layers of the network.
C_{in}^{l}	Input Channel	The number of input channels in l-th layer.
C_{out}^{l}	Output Channel	The number of output channels in l-th layer.
K_h, K_w	Kernel Spatial Dimensions	Height and width of the convolution kernel.
H_{out}^{l}, W_{out}^{l}	Output Feature Map Dimensions	Spatial resolution of the output feature map.
N_{active}	Active convolutional kernels	The number of active kernel columns.

6. Benefit of the APCNN and comparison with other neuromorphic hardware systems.

Table S2. Benefit across various pruning rounds for Fashion-MNIST dataset.

Round	No. of kernels	Accuracy (%)	Params	MACs	P_{array} (μW)	$P_{\mathrm{infer.core}}$ (μW)	Energy per VMM (nJ)
Pre-Train	96	92.03	334,168	4,153,064	428.76	1394.52	6.97
1	58	91.30	227,540	1,345,168	124.70	708.18	3.54
2	35	90.42	135,859	570,772	46.97	399.07	2.00
3	25	90.21	90,890	360,856	28.06	297.56	1.49
4	18	90.11	60,960	229,144	15.90	196.98	0.985
5	15	90.28	46,083	182,692	12.20	163.10	0.82
6	12	90.05	36,088	132,320	7.76	128.48	0.64
7	11	90.01	31,138	118,600	6.79	117.45	0.59
8	10	89.88	26,188	104,880	5.64	106.24	0.53

Table S3. Comparison with other neuromorphic hardware systems.

Device	Node	Precision	Power (μW)	Efficiency	Evaluation boundary	Reference
FeFET	28 nm	2-bit input/2-bit weight	153.6	885.4 TOPS/W	Crossbar and ADCs	[4]
1T1R RRAM	130 nm	Configurable	/	Up to 49.7 TOPS/W	VMM-core metric and neuron/ADC	[5]
PCM	14 nm	8-bit input/output	/	2.48-9.76 TOPS/W	64-core chip	[6]
Fe-AmFET	5-μm gate length	7-bit weights	106.24	0.531 nJ/VMM	Array and analog D-SH	This work

Notably, given large discrepancies across reported works in terms of technology node, computation precision, and whether peripheral circuits are accounted for, this comparison is intended to clarify the operating range and evaluation boundary rather than claim superiority.

Note S3. Influence of hardware non-idealities on kernel pruning decisions and network performance.

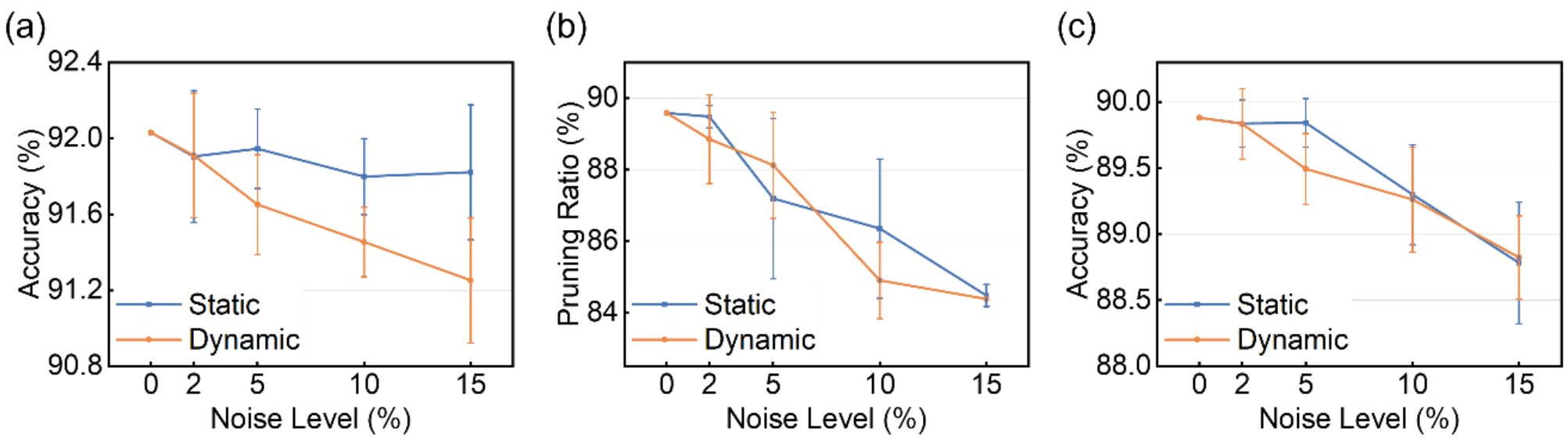

Figure S4. Influence of hardware non-idealities on kernel pruning decisions and network performance. (a) Pre-training accuracy, (b) pruning ratio, and (c) final post-pruning accuracy under different Gaussian noise levels. All results are evaluated on the Fashion-MNIST dataset and averaged over ten independent trials.

For practical deployment, the hardware inevitably suffers from device-to-device variation, temporal fluctuation, and programming noise. To address this issue, we analyze their influence on kernel pruning decisions and overall network performance. Specifically, we classify hardware influence into two physically distinct categories, static noise and dynamic noise.

Static Noise stems from device-to-device variations and weight programming uncertainty, which induces deviations between the practically implemented weights and the theoretical weights. Such non-idealities remain unchanged throughout the entire network training, pruning, and inference process. Here we model it as a Gaussian perturbation,

$$w_{ij}^{stat} = w_{ij}\left(1 + \varepsilon_{ij}^{stat}\right), \quad \varepsilon_{ij}^{stat} \sim N(0, \sigma_{stat}^2),$$

where w_{ij} is the theoretical weight of the Fe-AmFETs, w_{ij}^{stat} is the weight with static noise, and σ_{stat} is the static noise level.

Dynamic Noise corresponds to temporal fluctuations. It is introduced during both the pruning process and inference process to emulate real-time fluctuations in analog hardware, which is modeled as a Gaussian perturbation as,

$$w_{ij}^{dyn}(t) = w_{ij}\left(1 + \varepsilon_{ij}^{dyn}(t)\right), \quad \varepsilon_{ij}^{dyn}(t) \sim N(0, \sigma_{dyn}^2),$$

where w_{ij} is the theoretical weight of the Fe-AmFETs, $w_{ij}^{dyn}(t)$ is the weight with the dynamic noise $\varepsilon_{ij}^{dyn}(t)$ that varies with the operating time *t*, and σ_{dyn} is the dynamic noise level.

To analyze the influence of hardware non-idealities on kernel pruning decisions and overall network performance, we perform evaluations on the Fashion-MNIST dataset following the same pre-training/pruning/fine-tuning workflow as described in the main text. All results are averaged over ten independent repeated trials to ensure statistical credibility. Figure S4 displays the pre-training accuracy, pruning ratio, and final post-pruning accuracy under various static and dynamic noise levels. It can be seen that these metrics show slight degradation as the noise level increases. Although these noises may slightly affect the importance scoring of a small number of marginal kernels, the overall pruning decisions are not significantly changed. Accordingly, the pruned network retains almost consistent in inference accuracy within the tested noise range. These demonstrate that the Fe-AmFET-based adaptive pruning scheme has favorable robustness against device-to-device variation, temporal fluctuation, and programming noise.

Note S4. Performance evaluation of the adaptive pruning convolutional neural network on ResNet.

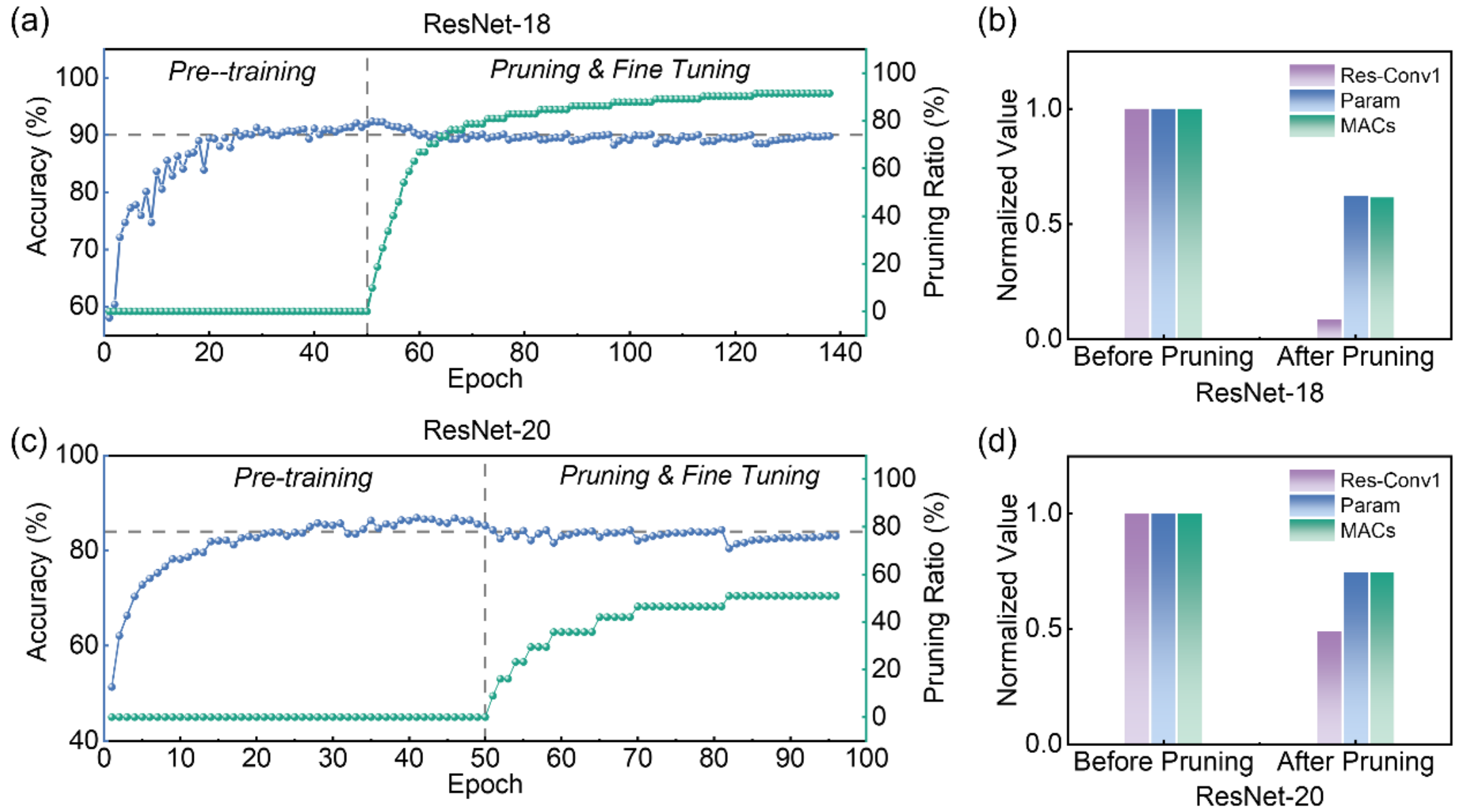

Figure S5. Performance evaluation of the adaptive pruning convolutional neural networks based on ResNet-18 and ResNet-20 on the CIFAR-10 dataset. (a) Relationship between the inference accuracy and pruning ratio for ResNet-18. (b) Pruning benefits for ResNet-18. (c) Relationship between the inference accuracy and pruning ratio for ResNet-20. (d) Pruning benefits for ResNet-20. To demonstrate the scalability and verify whether similar benefits can be achieved in more realistic scenarios, we deploy ResNet-18 and ResNet-20 on our Fe-AmFET hardware framework and conduct evaluations on the CIFAR-10 dataset. Each residual block of ResNet contains two convolutional layers. To guarantee structural integrity after pruning and prevent dimension mismatch in residual shortcut connections, we only perform kernel pruning on the first convolutional layer while keeping the output dimension of each residual block unchanged. As displayed in Fig. S5, the results confirm that our device-native pruning mechanism works on much deeper, more complex networks. The baseline classification accuracy of unpruned ResNet-18 and ResNet-20 on CIFAR-10 is 92.11% and 86.79%, respectively. After adopting our Fe-AmFET-based pruning scheme, ResNet-18 attains a kernel pruning ratio of 91.46% (from 1920 to 164), accompanied by a 37.64% reduction in parameters (from 11.16 M

to 6.96 M) and a 38.47% drop in MAC operations (from 555.42 M to 341.75 M), with only a 2.22% accuracy degradation. For ResNet-20, our method delivers a 50.89% kernel pruning ratio (from 336 to 165), alongside 25.36% fewer parameters (from 0.27 M to 0.20 M) and a 25.34% reduction in MAC operations (from 40.81 M to 30.47 M), at an accuracy loss of only 3.68%.

Table S4. Comparison of different CIM hardware for neural network performance.

	FeFET	1T1R RRAM	PCM	This work	
Dataset	CIFAR-10	CIFAR-10	CIFAR-10	CIFAR-10	
Network Architecture	VGG19	ResNet-20	ResNet-9	ResNet-18	ResNet-20
Accuracy	91.55%	85.7%	92.81%	92.1%	86.8%
Pruning Ratio	/	/	/	91.46%	50.89%
Accuracy after Pruning	/	/	/	89.89%	83.11%
Reference	[4]	[5]	[6]	This work	

Table S4 indicates that our architecture achieves performance comparable to FeFET-, 1T1R RRAM-, and PCM-based hardware neural networks. Nevertheless, none of these hardware implementations have built-in pruning functionality.

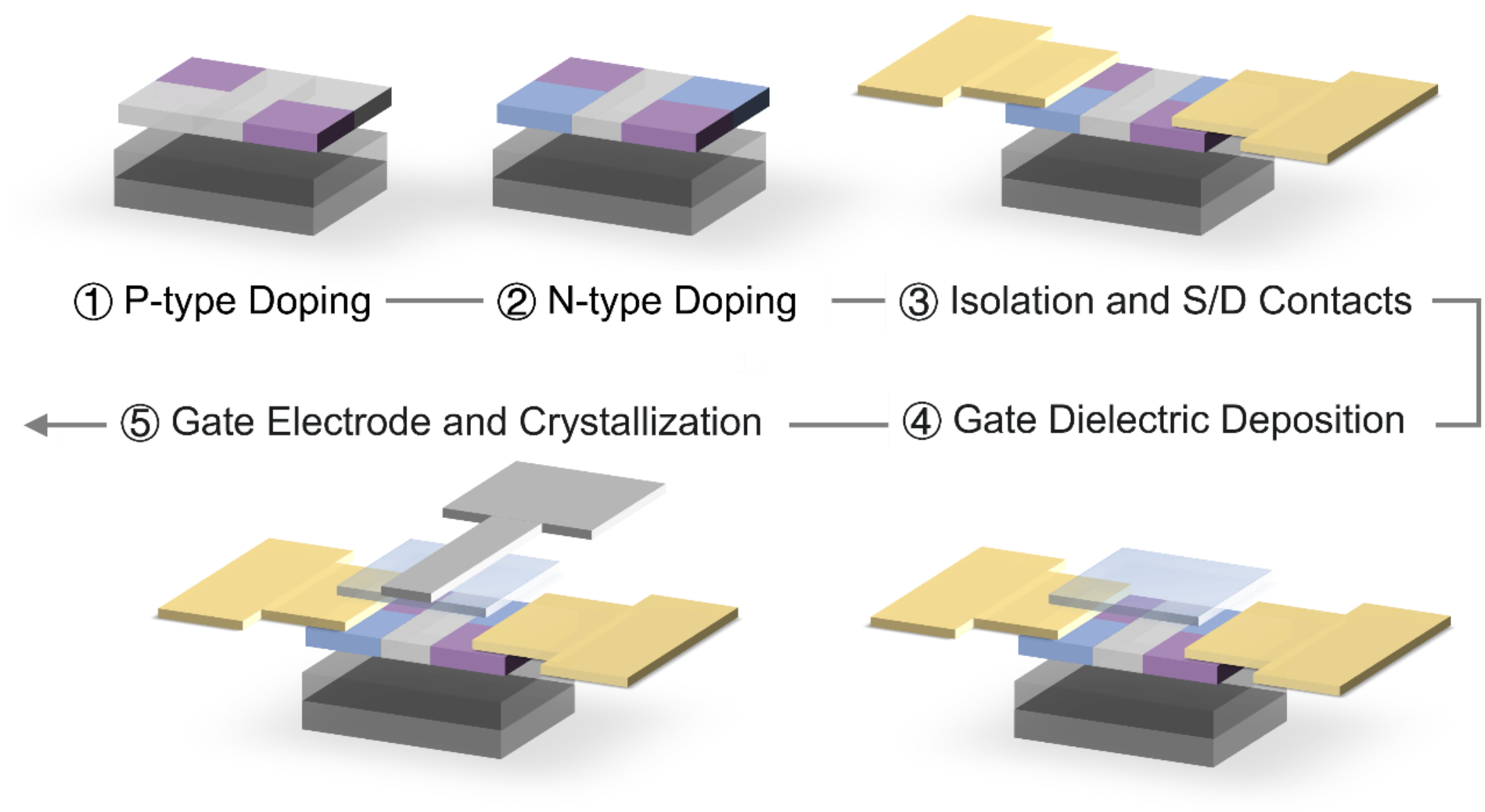

Figure S6. Fabrication process of the Fe-AmFETs. (a) Boron ion implantation is conducted to define the p^{++}-doped subregions (b) Phosphorus ion implantation is performed to form the n^{++}-doped subregions. (c) Active areas of the devices are isolated, followed by the deposition and patterning of the source and drain metal electrodes. (d) Deposition of the $Hf_{0.5}Zr_{0.5}O_2$ (HZO) ferroelectric dielectric film. (e) Tungsten (W) top gate electrode is deposited, followed by a thermal annealing process to induce ferroelectric crystallization in the HZO layer.

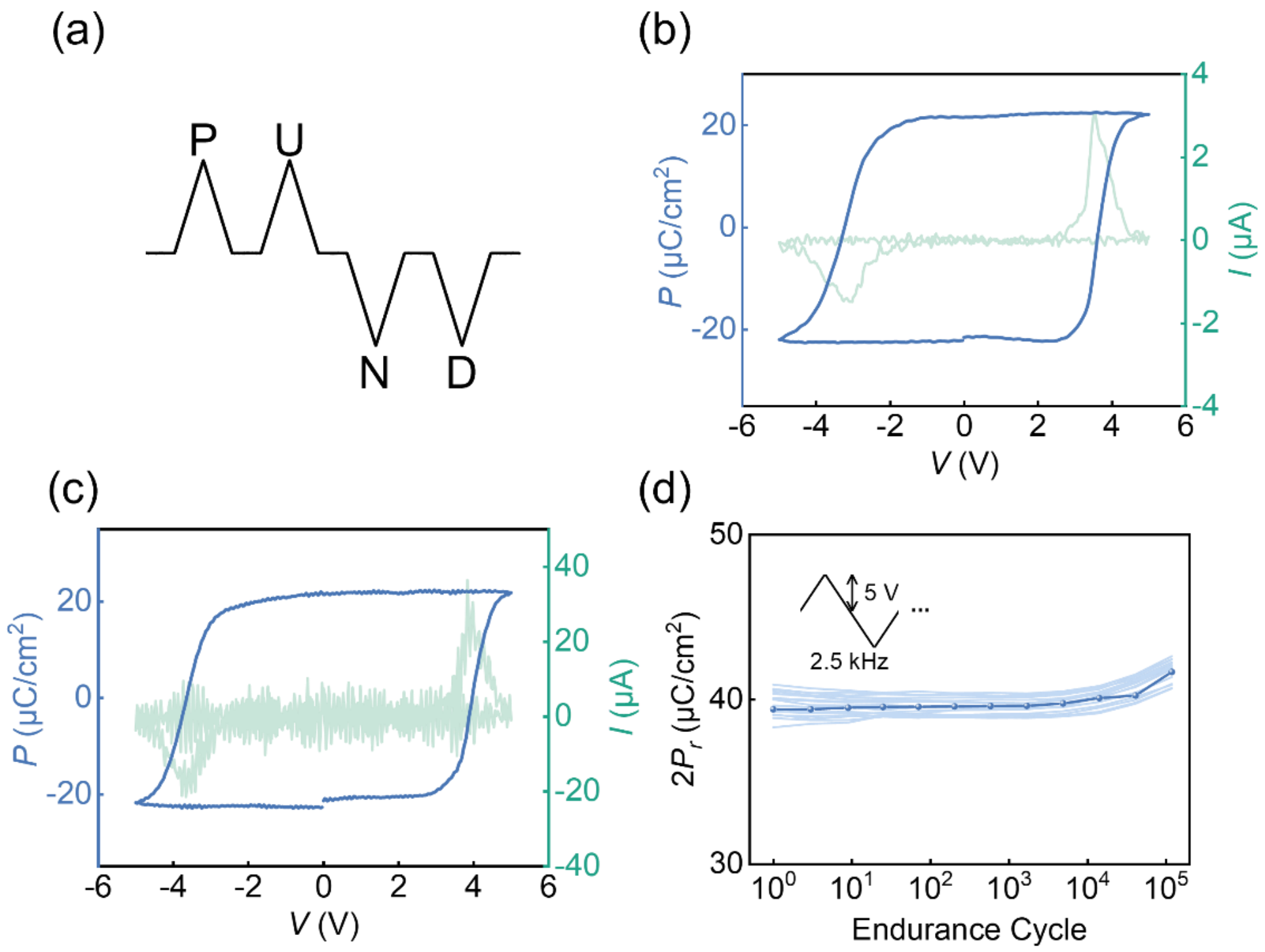

Figure S7. Ferroelectric characteristics of the HZO film. (a) Schematic of the positive-up-negative-down (PUND) measurement protocol, which is used to extract the intrinsic remanent polarization by suppressing contributions from non-ferroelectric leakage. For the characterization, the transistor gate serves as one electrode, while the shorted source and drain (S/D) terminals act as the counter electrode. (b) and (c) Polarization-voltage (blue) and current-voltage (green) loops under a frequency of 2.5 kHz and 25 kHz, respectively. (d) Endurance characteristics of the HZO film under fatigue cycling with ±5 V pulses (test frequency, 2.5 kHz), which are randomly acquired from 15 independent devices with the average value displayed by the highlighted line. As shown in Fig. S7b and Fig. S7c, the device exhibits a considerable remanent polarization (2*P*r) of 43.1 $\mu C/cm^2$ at a modulation frequency of 2.5 kHz. Even when the frequency is increased to 25 kHz, the 2*P*r value is well retained at 42.8 $\mu C/cm^2$. In addition, the statistic in Fig. S7d exhibits that the devices maintain the high level of remanent polarization after 10^5 endurance cycles with a good device-to-device uniformity. Notably, all ferroelectric characterizations in this work are performed without additional electrical wake-up pretreatment. For HZO-based ferroelectrics, electrical wake-up cycling facilitates the redistribution of

mobile defects, especially oxygen vacancies. This defect rearrangement alleviates domain wall pinning and suppresses built-in bias fields, which in turn increases remanent polarization and stabilizes device performance during repetitive cycling. The requirement for wake-up pretreatment is dependent on multiple factors, such as HZO film stacking structure, electrode materials, interfacial quality, intrinsic defect density, and overall fabrication procedures. Our Fe-AmFET features a W/HZO/SiO_2/Si gate stack. Following the deposition of the tungsten top electrode, the devices undergo annealing at 500 ºC for 30 s under N_2 ambient. This optimized thermal treatment sufficiently activates robust, stable ferroelectricity in the HZO gate stack, as verified by the polarization switching responses from PUND tests (Fig. S7b and Fig. S7c) and well-defined memory windows (Fig. 2c) of the as-annealed devices. Accordingly, all ferroelectric and synaptic characteristics reported in this work can be acquired without any electrical wake-up pretreatment.

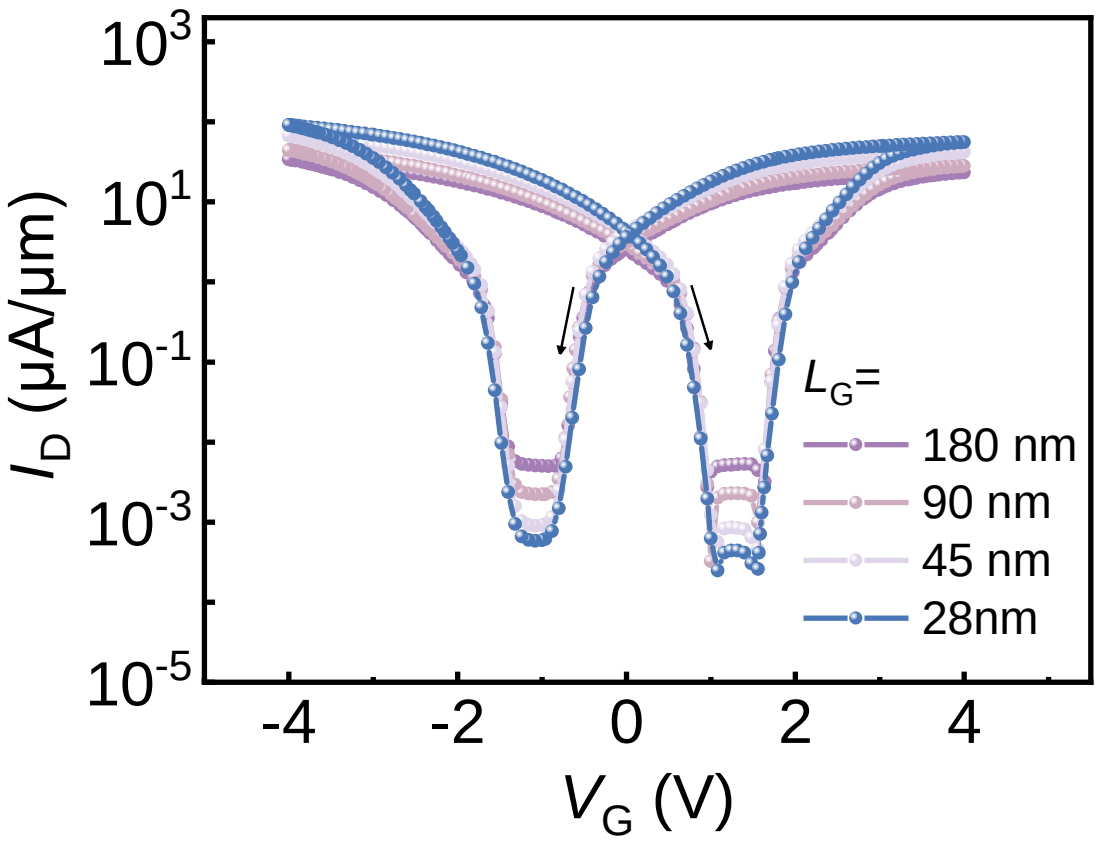

Figure S8. Evaluation of the miniaturization capability of the Fe-AmFETs via TCAD simulations. To explore the potential of the devices in advanced Si processes, TCAD simulations are performed on the Fe-AmFETs with a fixed width-to-length ratio (W/L=4), with the ferroelectric HZO layer modeled with a coercive field E_C=2 MV/cm and a remanent polarization P_r=20 μC/cm^2. The thicknesses of the HZO and SiO_2 films are 12 nm and 1 nm, respectively, which are similar to those in the experiment. As shown in the results, even when the channel length is scaled down to 28 nm, the device maintains remarkable ON/OFF current ratios for both n-type and p-type branches. This indicates that the complementary ambipolar ohmic contact remains effective for efficient electron and hole transport. In addition, the memory window degrades negligibly as channel length shrinks. Therefore, it can be concluded that the Fe-AmFET has potential in scalability. Since weight resolution relies on the separability of discrete programmed conductance states, these simulation results further indicate that multilevel weight storage can be realized in miniaturized Fe-AmFET devices.

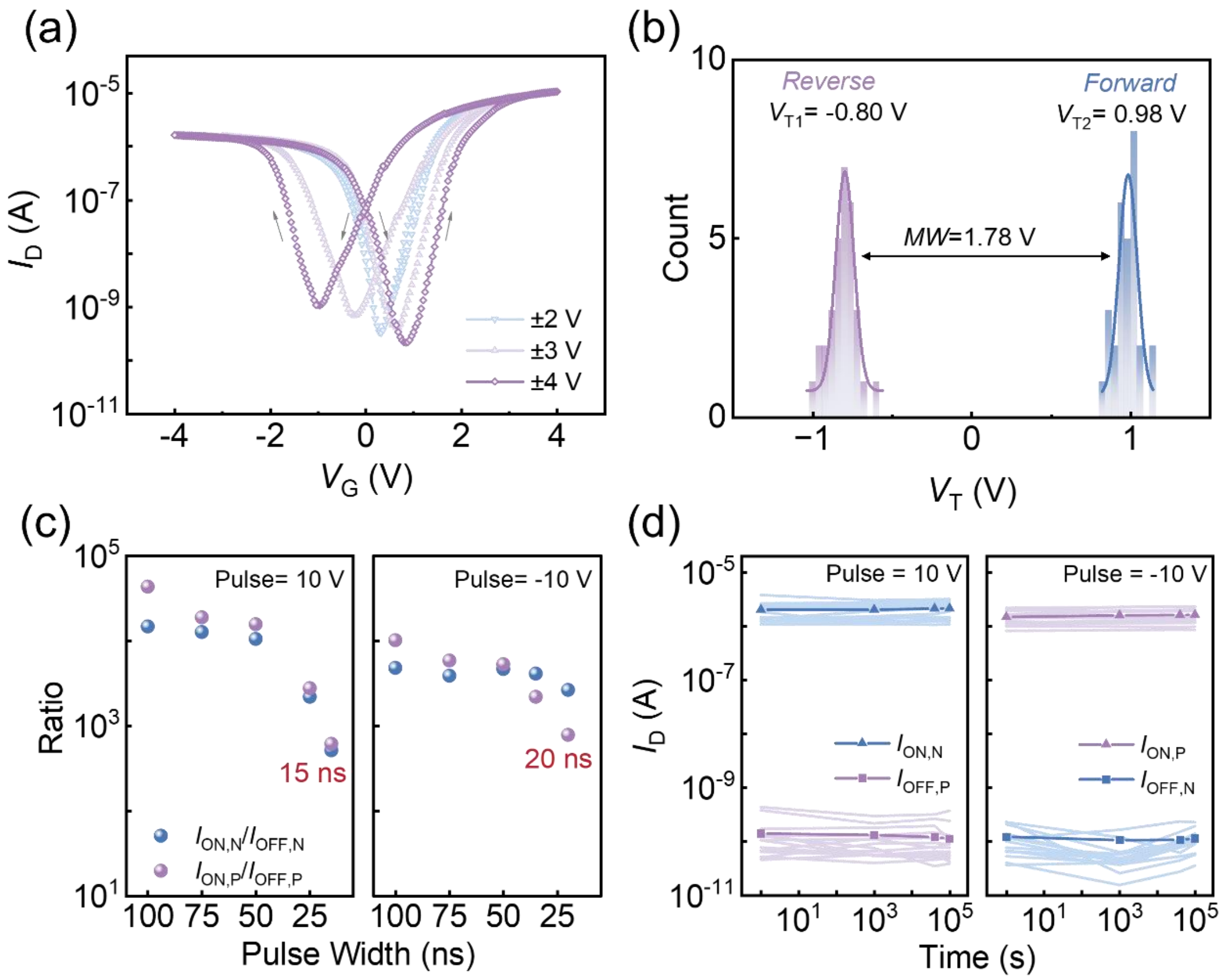

Figure S9. Electrical characteristics of the Fe-AmFETs. (a) Looped transfer characteristic curves of the Fe-AmFET under various gate voltage sweep ranges, demonstrating an obvious ferroelectric memory window (MW) that expands with increasing sweep amplitude. (b) Threshold voltage (V_T) shift and memory window statistic across 30 devices. The MW is defined as the threshold voltage shift between forward and backward sweeps, demonstrating that the average MW is ~ 1.78 V at a maximum sweeping voltage of 4 V. (c) Pulse-width-dependent ON/OFF ratios for n-mode and p-mode operations under ±10 V gate pulse voltages. The devices maintain a current ON/OFF ratio of ~10^3 even when the pulse widths are reduced to 15 ns (programming) and 20 ns (erasing), respectively, indicating ultrafast programming/erasing speeds. (d) Retention performances of the Fe-AmFETs, which are randomly acquired from 15 independent devices with the average value displayed by the highlighted line. The statistic indicates that the drain currents exhibit negligible decay after 10^5 s after the application of programming/erasing gate pulse voltages with a favorable device-to-device uniformity.

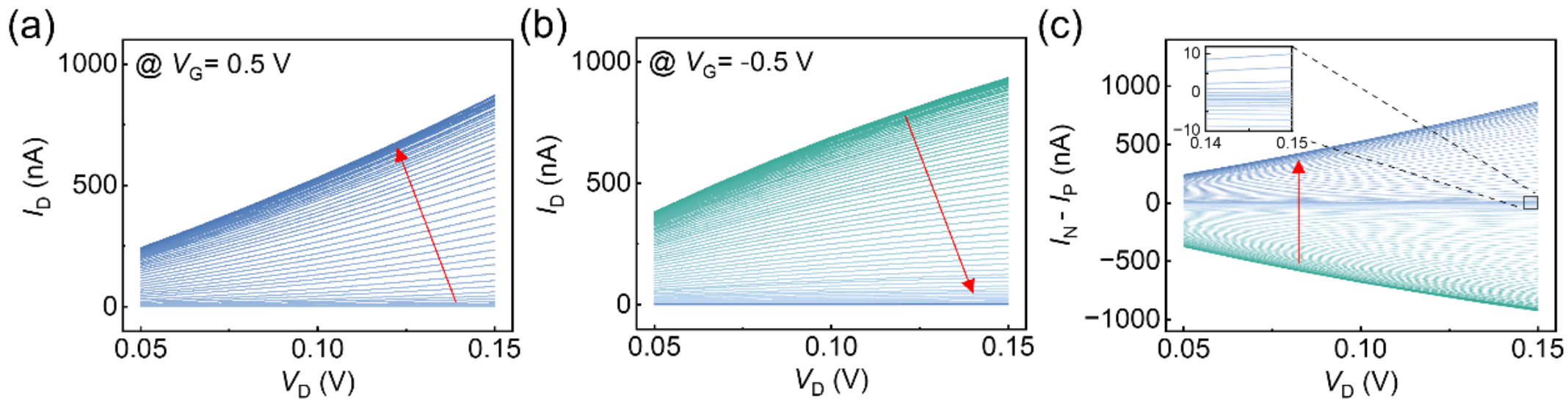

Figure S10. Output characteristic curves under various weight states. I_D-V_D curves of the Fe-AmFET programmed with different gate pulses, which are measured at a gate bias of (a) V_G=0.5 V and (b) V_G=-0.5 V, respectively. (c) Differential readout current curves derived from the n- and p-mode responses in (a) and (b). The devices exhibit excellent robustness, with 128 well-resolved output levels corresponding to a 7-bit effective weight resolution.

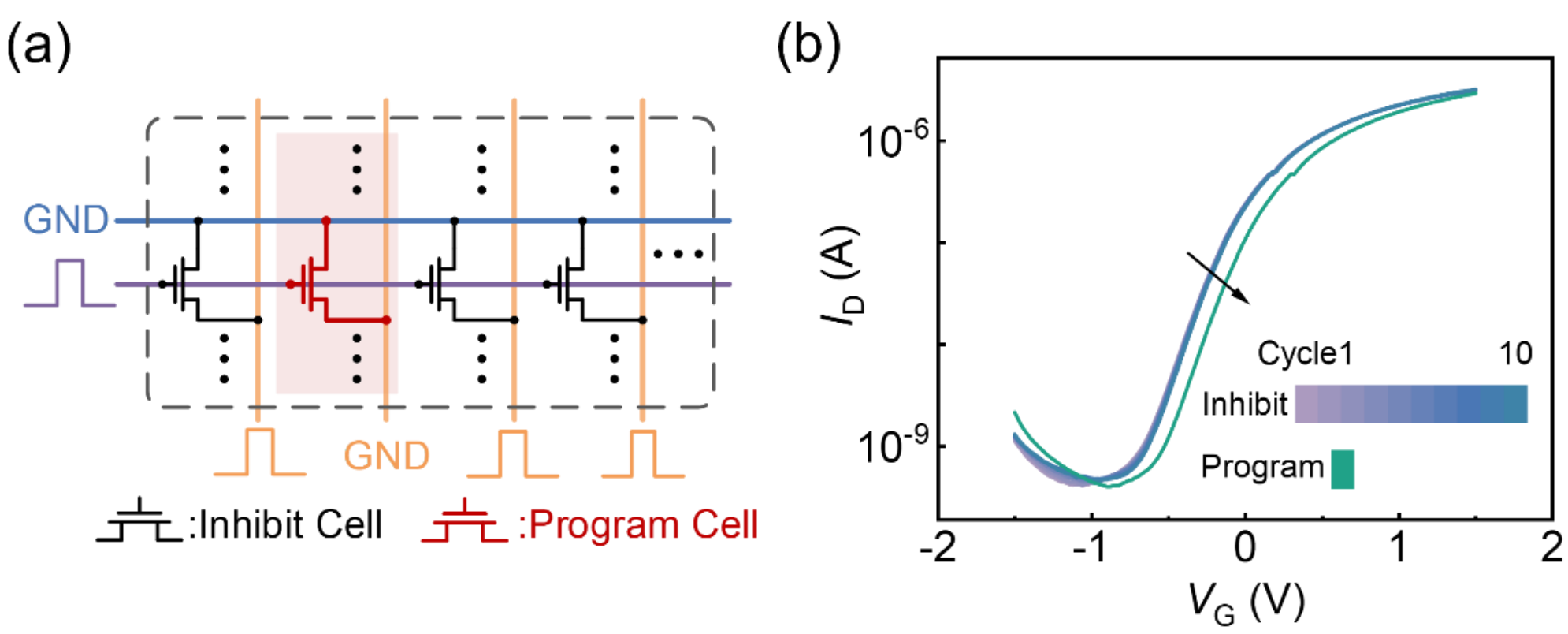

Figure S11. Weight programming scheme in the Fe-AmFET crossbar array. (a) Schematic diagram of the weight programming scheme. By applying specific voltage pulses to the intersecting word lines (WLs) and source lines (SLs) with the bit lines (BLs) grounded, the polarization direction of each Fe-AmFET can be modulated and its weight can be tuned. When the SL corresponding to a specific device is grounded (SL=0 V), the electric field across the gate-channel interface exceeds the coercive field (E_c) of the HZO ferroelectric layer, enabling reliable programming. In contrast, when synchronized programming pulses are applied to both the SL and WL, the gate-to-source voltage is reduced to near zero, such that the resulting electric field is insufficient to switch the ferroelectric polarization, thus effectively suppressing programming in unselected devices. (b) Experimental validation of the programming inhibit scheme. To evaluate the write-disturbance robustness, a high-amplitude programming pulse (10 V, 100 ns) is applied, which greatly exceeds the standard programming voltage. Even after 10 consecutive programming pulses, the transfer characteristics show a negligible threshold voltage (V_T) shift, confirming that the ferroelectric polarization remains undisturbed. By comparison, when the source is grounded under the same gate pulse, the ferroelectric polarization switches effectively, leading to a shift in the transfer curves.

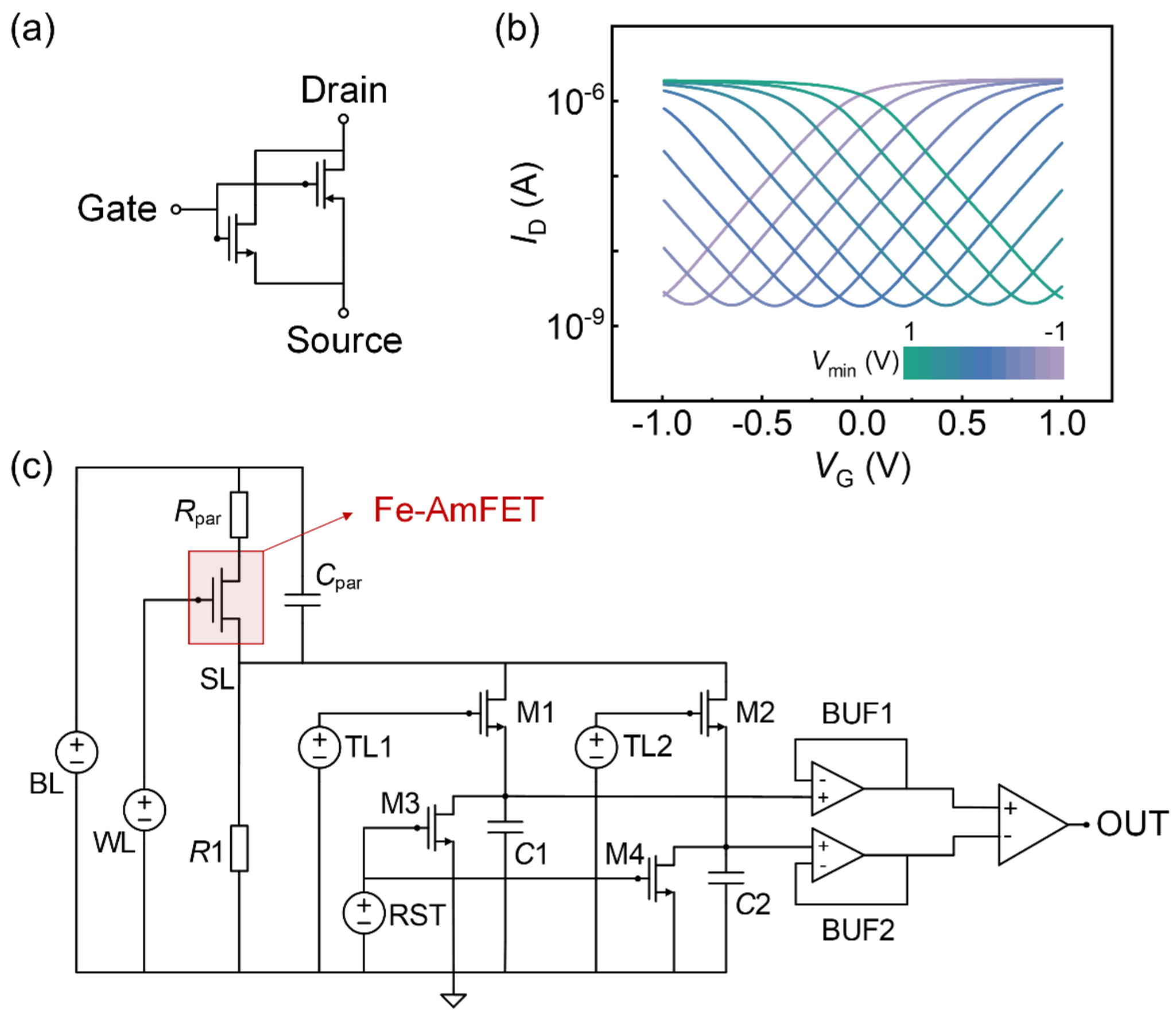

Figure S12. SPICE simulations on the Fe-AmFET and D-SH readout circuit. (a) Device model of the Fe-AmFET. As no standard compact model for the Fe-AmFET is available in conventional SPICE libraries, we employ an equivalent circuit composed of two parallel-connected NMOS and PMOS transistors to reproduce the intrinsic ambipolar transport characteristics. A tunable parameter V_{min} is introduced to model the equivalent electrostatic doping effect in the channel from different ferroelectric polarization states. (b) SPICE-simulated I_D-V_G curves of the Fe-AmFET under different programming states. (c) Schematic of the D-SH readout circuit. The timing control signals for the BL (bit line), WL (word line), TL1, TL2, and RST follow the sequence shown in Fig. 3d. BUF1 and BUF2 serve as voltage followers (buffers) to electrically isolate the sampling stage from the differential processing stage and enhance the driving capability for subsequent circuitry. To focus on validating the feasibility of the readout circuit itself while preserving the practical load condition of the array, we simplify the full array into a single representative Fe-AmFET device. This simplification does not degrade signal integrity and is sufficient for circuit-level

verification. Detailed SPICE simulation parameters are summarized in Table S5.

Table S5. SPICE simulation parameters for the readout circuit.

Parameter	Value	Description
Sampling pulse width	1 μs	Width of the word line (WL) activation signal
$C1$&$C2$	10 pF	Sampling capacitors of the D-SH circuit
R_{par}	1 kΩ	Estimated interconnect parasitic resistance (representing worst-case IR drop)
C_{par}	10 fF	Parasitic capacitance
$R1$	1 kΩ	Precision resistor for current-to-voltage conversion at the source line (SL)

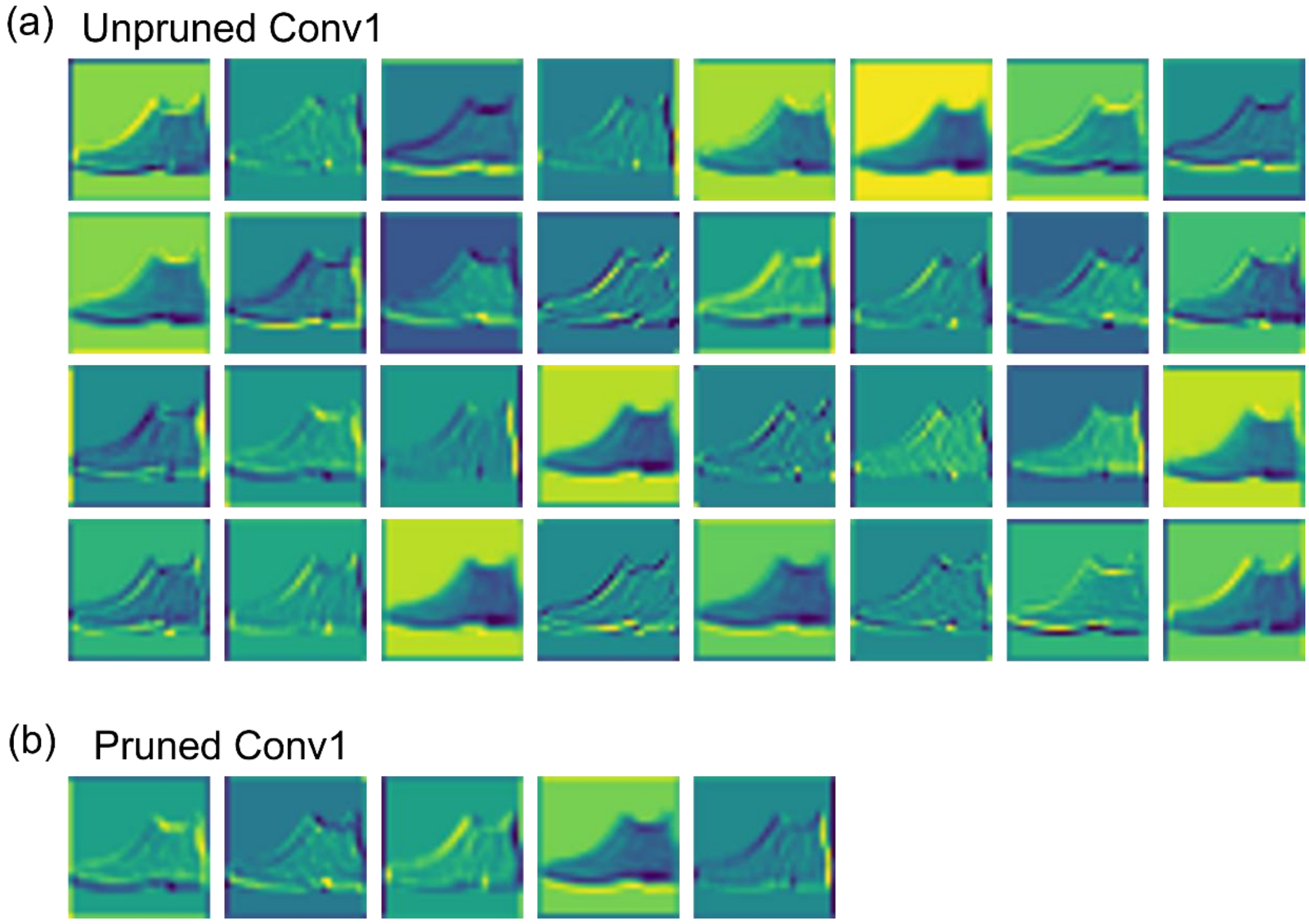

Figure S13. Feature maps before and after pruning for the Fashion-MNIST dataset. (a) 32 feature maps extracted by the Conv1 layer of the unpruned network. Before pruning, the Conv1 layer exhibits significant redundancy. Many convolution kernels produce highly correlated feature maps, where only one kernel is enough for specific feature extraction. In addition, some kernels generate blurred and invalid feature maps that contribute little to effective feature extraction and fail to provide useful feature maps. (b) Remaining feature maps after pruning. The remaining feature maps mainly capture salient structural information and distinct edges, demonstrating that the network maintains effective feature extraction and classification capability even with substantially fewer kernels.

REFERENCES

1. Molchanov P, Tyree S, Karras T *et al*. Pruning convolutional neural networks for resource efficient inference. In: *International Conference on Learning Representations* (*ICLR*), 24-26 April, 2017. OpenReview.net, Toulon, France.
2. Molchanov P, Mallya A, Tyree S *et al*. Importance estimation for neural network pruning. In: *2019 IEEE/CVF Conference on Computer Vision and Pattern Recognition (CVPR)*, 16-20 June 2019. pp. 11256-64. IEEE, Long Beach, CA, USA.
3. He Y, Dong X, Kang G *et al*. Asymptotic soft filter pruning for deep convolutional neural networks. *IEEE Trans Cybern* 2020; **50**: 3594-604.
4. Soliman T, Chatterjee S, Laleni N *et al*. First demonstration of in-memory computing crossbar using multi-level cell FeFET. *Nat Commun* 2023; **14**: 6348.
5. Wan W, Kubendran R, Schaefer C *et al*. A compute-in-memory chip based on resistive random-access memory. *Nature* 2022; **608**: 504-12.
6. Le Gallo M, Khaddam-Aljameh R, Stanisavljevic M *et al.* A 64-core mixed-signal in-memory compute chip based on phase-change memory for deep neural network inference. *Nat Electron* 2023; **6**: 680-93.
